# Visualization of microRNA therapy in cancers delivered by small extracellular vesicles

**DOI:** 10.1186/s12951-023-02187-5

**Published:** 2023-11-29

**Authors:** Peiwen Fu, Yumeng Guo, Yanan Luo, Michael Mak, Jianguo Zhang, Wenrong Xu, Hui Qian, Zhimin Tao

**Affiliations:** 1 Jiangsu Province Key Laboratory of Medical Science and Laboratory Medicine, Department of Laboratory Medicine, School of Medicine, Jiangsu University, Zhenjiang, 212013 Jiangsu China; 2 Department of Laboratory Medicine, Nanjing First Hospital, Nanjing Medical University, Nanjing, 210006 Jiangsu China; 3 Department of Biomedical Engineering, School of Engineering and Applied Science, Yale University, New Haven, 06520 USA; 4 Department of Emergency Medicine, The Affiliated Hospital, Jiangsu University, Zhenjiang, 212001 Jiangsu China; 5 Zhenjiang Key Laboratory of High Technology Research on Exosomes Foundation and Transformation Application, School of Medicine, Jiangsu University, Zhenjiang, 212013 Jiangsu China

**Keywords:** Extracellular vesicles, microRNA, Quantum dots, Gene delivery, Cancer therapy

## Abstract

MicroRNA (miRNA) delivery by extracellular vesicles (EVs) has recently inspired tremendous developments in cancer treatments. However, hybridization between miRNA and its target mRNA is still difficult to be imaged in vivo to assess the therapeutic effects in time. Herein we design a nano-scale fluorescent “off–on” complex encapsulated by small extracellular vesicles (sEVs) for real-time visualization and evaluation of gene therapy efficiency in human gastric cancer cells and murine xenograft tumor models. The complex is formed by π–π stacking between graphene quantum dots (GQDs) and tumor suppressor miR-193a-3p conjugated fluorescent tag whose signals remain off when binding to GQDs. Loaded into sEVs using tunable sonication techniques, the GQDs/Cy5-miR particles enter the tumor cells and promote miR-193a-3p escape from endosomes. The miR-193a-3p in GQDs/Cy5-miR is unleashed to pair the specific target oncogene cyclin D1 (*CCND1*), therefore turning on the fluorescence of miRNA tags. We find out that GQDs/Cy5-miR@sEVs can activate the “turn-on” fluorescent signal and exhibit the longest retention time in vivo, which suggests a minimized degradation of miR-193a-3p in dynamic processes of miRNA-mRNA binding. More importantly, GQDs/Cy5-miR@sEVs significantly promote cancer apoptosis in vitro and in vivo via the enhanced cellular uptake. Our study demonstrates that GQDs/Cy5-miR@sEVs represent an efficient and refined theranostic platform for gene therapy in cancers.

**Graphical Abstract:**

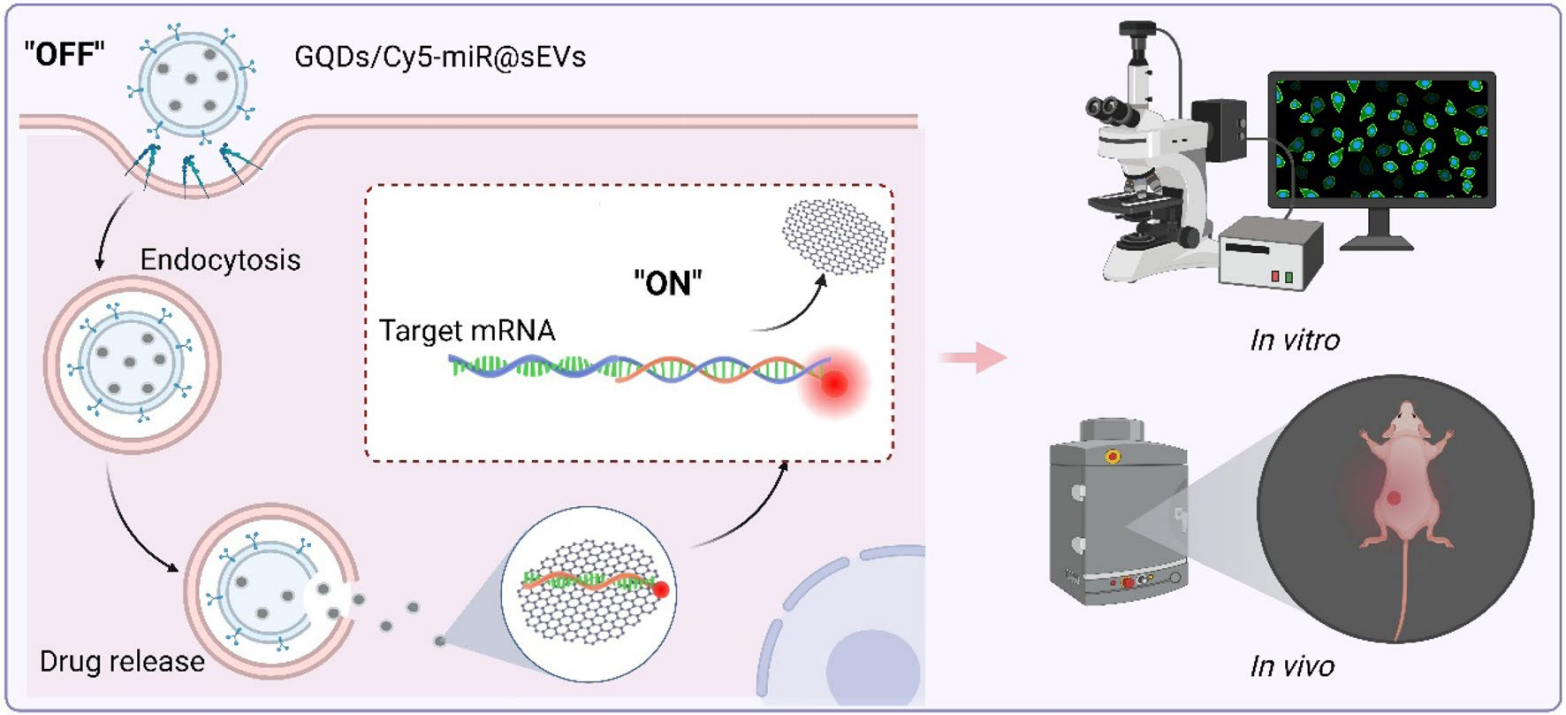

**Supplementary Information:**

The online version contains supplementary material available at 10.1186/s12951-023-02187-5.

## Introduction

Pharmaceutical nucleic acids have been long used in clinical and experimental medicine [1, 2]. Among them, small interfering RNA (siRNA), antisense oligonucleotides (ASO), microRNA (miRNA), mRNA and clustered regularly interspaced short palindromic repeats-associated protein 9 (CRISPR/Cas9) system exhibited active regulation or correction of gene expression in many disease therapeutics [3–5]. Among them, miRNAs can downregulate their target mRNAs through interactions with their 3′ untranslated regions (UTRs) [6]. Despite of those developments, a real-time versatile system that could effectively monitor and mission the miRNA delivery is urgently needed. On one hand, albeit several current assays, luciferase reporter gene system for example, might evaluate the miRNA-mRNA interactions, it is limited at the cellular level with low accuracy [7]. On the other hand, due to the poor loading of RNA molecules using traditional methods, such as electroporation, chemical transfection, and parent cell modification, how functional RNA cargo can be actively encapsulated and efficiently transferred to intracellular targets remains a challenge [8, 9]. Insofar, there is yet reliable and persistent evaluation system that reveals the dynamic miRNA-mRNA pairing in vivo.

Extracellular vesicles (EVs) are natural particles secreted by almost all cell types, indispensable in intercellular communications [10, 11]. Based on their size, EVs can be categorized as small EVs (sEVs, 50–200 nm) and medium or large EVs (> 200 nm) [12, 13]. Given their comparable dimension to many biomolecules, low immunogenicity, and unique natural origin, sEVs have been recognized as one of efficient delivery systems that transport a diversity of biological and pharmaceutical molecules [14]. Although several drug delivery methods (e.g., liposomes and hydrogel microspheres) have been widely adopted, many of these synthetic nanoparticles can be rapidly recognized and neutralized by the immune system after intravenous administration [15]. In comparison, sEVs were superior in tumor targeting and immune evading due to the highly expressed specific proteins on the surface, such as lipocalin-2 (LCN2) and tetraspanin protein CD47 [16, 17]. With low carcinogenicity and non-immunogenicity, sEVs derived from human umbilical cord mesenchymal stem cells (hucMSCs) inherit the therapeutic function of the parent hucMSCs while obviating their biosafety concerns [14, 18]. Simultaneously, there is mounting evidence that EVs can transfer their luminal cargo (e.g., RNA) into the cytosol of recipient cells, showing their good capability of escaping the endolysosomal pathway [19]. For these biological features, and with rapidly developing technology on analyzing EVs [20], sEVs hold great potential as the next-generation delivery platform [21].

**Fig. 1:**
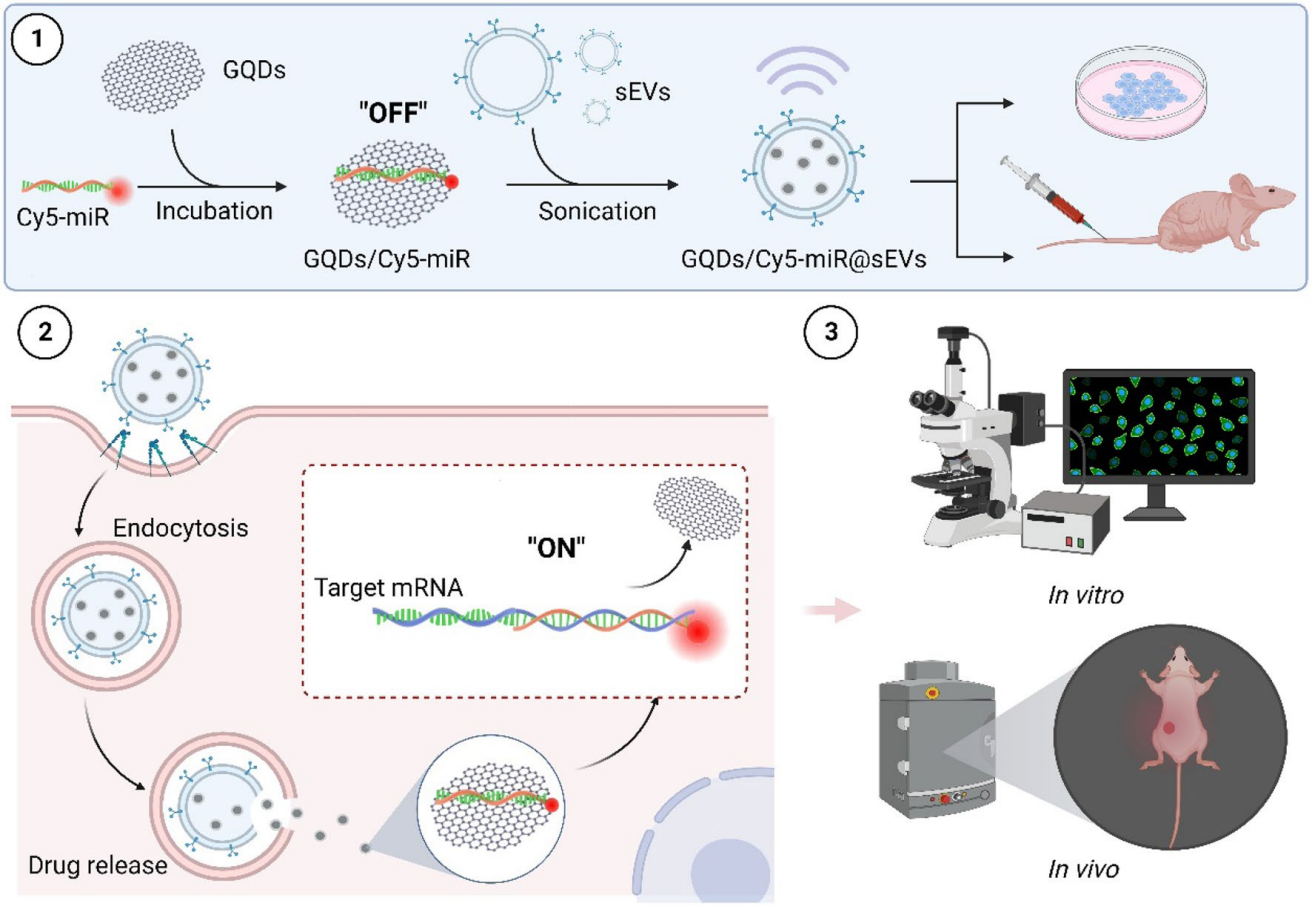
Illustrations that delineate the construct of GQDs/Cy5-miR@sEVs and their applications in the biological systems. (1) The miRNA with chemical modification of Cy5 was attached to GQDs via “π–π” stacking in “off” state, followed by the encapsulation into sEVs by sonication and further delivery into cells or animals. (2) The miRNA encapsulated into GQDs/Cy5-miR@sEVs was delivered into cells via endocytosis, released inside the cytoplasm, and hybridized with the targeted mRNA to restore the quenched fluorescence (leading to the “on” signal). (3) The GQDs/Cy5-miR@sEVs were tested in cancer cells and tumor-bearing animal models, to visualize gene therapy of the delivered miRNA both in vitro and in vivo

In this study we devise an sEVs-based theranostic nanoplatform that works to deliver and visualize the miRNA therapeutics in a murine model of xenografted human gastric cancer (GC). GC is one of the most malignant tumors with high metastatic potential, where the five-year overall survival rate of patients is < 10% [22]. This constructed system in nano dimension is primarily comprised of fluorescence-tagged miRNA in complexes with graphene quantum dots (GQDs), further loaded by sEVs that originate from hucMSCs). As graphene is a one-atom thick sheet of *sp*^2^ carbon network, it interacts with the aromatic ring of fluorescent tag that links to miRNA through π–π stacking [23], efficiently quenching the fluorescence of carboxyl fluorescein (or fluorescein amidite, FAM) or cyanine 5 (Cy5) in the miRNA tags. When miRNA is hybridized with its corresponding target gene to form a duplex by complementary base pairing, the duplex detaches from the surface of GQDs. This leads to the recovery of fluorescence, attaining visibility during miRNA treatment in vivo (Fig. 1).

## Experimental section

### Cell culture

HucMSCs were cultured in alpha minimum essential medium (α-MEM, Invitrogen, USA) with 10% fetal bovine serum (FBS, Gibco, USA). Human gastric cancer cells (HGC-27) were purchased from Institute of Biochemistry and Cell Biology at the Chinese Academy of Sciences (Shanghai, China) and maintained in Dulbecco's Modified Eagle's Medium (DMEM) containing 10% FBS at 37 °C with 5% CO_2_.

### Isolation and characterization of hucMSCs and their derived sEVs

We isolated and cultured hucMSCs as previously reported [24]. Briefly, MSCs were isolated from human umbilical cord, as the primary MSCs were cultured in α-MEM with 10% FBS. The human umbilical cords were obtained from Affiliated Aoyang Hospital of Jiangsu University. The experimental protocol was approved and informed consent from patients was waived by the Ethics Committee at Affiliated Aoyang Hospital of Jiangsu University. MSCs were passed once for further experiments, and sEVs were isolated as previously reported [24]. The serum-free DMEM medium supernatant of hucMSCs was centrifuged at 2000×*g* for 30 min to remove cell debris. Then, the supernatant was centrifuged at 10,000×*g* for 30 min to remove organelle fragment. ZetaView PMX120 (Particle Metric, Germany) was applied to measure and analyze the particle size and zeta potential.

### Synthesis and characterization of GQDs/Cy5-miR@sEVs

Previous studies have confirmed that has-miR-193a-3p is a potent tumor suppressor miRNA and it targets various key oncogenic pathways across several cancer types, including human gastric cancer [25]. All Cy5-miR mimics, miRNA mimics and nonsense control (NC) mimics used in the study were synthesized by GenePharma China. The sequences of miRNA-193a-3p were 5′ AACUGGCCUACAAAGUCCCAGU 3′. The synthetic miRNA comprised of 21-base pair nucleotides, where the residual phosphate group of nucleotides was modified with fluorescent dyes (i.e., FAM or Cy5). Next, 20 µg GQDs (XFNANO, China) were mixed with 100 pmol (0.66 µg) of Cy5-miR, or 60 µg GQDs were mixed with 100 pmol (0.66 µg) of FAM-miR, in 30 µL RNase-free Tris-HCl at 37℃ for 30 min, to prepare the GQDs/Cy5-miR or GQDs/FAM-miR complexes. Please note that although Cy5 conjugates were primarily used, FAM tagged miRNA are alternatively applied when the Cy5 fluorescent channels were simultaneously needed for other labelling/tracking. GQDs/Cy5-miR@sEVs were prepared by loading GQDs/Cy5-miR into sEVs. Specifically, 30 µL GQDs/Cy5-miR were mixed with 20 µL sEVs (8 mg/mL; containing ~ 0.1 mg sEVs), followed by probe sonication for 3.5 min (5s on/10s off per cycle, 6 cycles, followed by 2 min on ice) twice at 25% power (output power 250 joules) by Ultrasonicator (SONICS VCX800, USA) to finally encapsulate GQDs/Cy5-miR into sEVs. Following the above steps, the mixture was through 10,000×*g* ultracentrifugation for 3 h to remove unincorporated free-floating Cy5-miR, GQDs and GQDs/Cy5-miR. So prepared GQDs/Cy5-miR@sEVs were immediately used for experiments.

### Western blotting

Total proteins from cells and tumor tissues were obtained by using radioimmunoprecipitation assay lysis buffer (Beyotime, China) that contains protease inhibitor cocktail. Then, Western blot analyses were performed as follows. Equal amounts of proteins from each group were separated by sodium dodecyl sulfate-polyacrylamide gel electrophoresis (SDS-PAGE) and then transferred onto polyvinylidene difluoride (PVDF) membranes. After blocking with 5% of nonfat milk for 1 h, the PVDF membranes were incubated with primary antibodies against a variety of proteins including Alix, CD63, CD9, calnexin (1:500, Cell Signaling Technology, USA), cyclin D1 (1:1000, Abcam, USA), and β-actin (1:10,000, Abcam, USA) overnight at 4 °C. Subsequently, the PVDF membranes were incubated with horseradish peroxidase (HRP)-labeled secondary antibodies (Vazyme, China).

### Quantitative real-time PCR (qRT-PCR) analysis

Total miRNA in the sEVs and GQDs/Cy5-miR@sEVs were isolated by using Exosome RNA Purification Kit (Qiagen, Germany), and the reverse transcription reactions of miRNA were performed using the 4×Reverse Transcription Master Mix kit (Qiagen, Germany). Subsequently, the qRT-PCR analysis of miRNA was carried out with QuantStudio^™^ 3 Real-Time PCR Instrument (Thermo Fisher Scientific, USA). The sequences of gene-specific primers were as follows:

1. CCND1: 5′-CCGAGAAGCTGTGCATCTAC-3′ (sense) and 5′-CTTCACATCTGTGGCACAGAG-3′ (antisense) (Sangon Biotech, China);
2. YWHAZ: 5′-CTCTCTTGCAAAGACAGCTTTT-3′ (sense) and 5′-GTCCACAATGTCAAGTTGTCTC-3′ (antisense) (Sangon Biotech, China);
3. miR-193a-3p: MS00031542 (Qiagen, Germany).

### Agarose gel electrophoresis assay

For the RNase stability test, Cy5-miRNA, GQDs/Cy5-miR and GQDs/Cy5-miR@sEVs (1 µM miRNA equivalent) were incubated at 37 °C in PBS containing RNase (1 mg/mL) for 30 min. The samples were analyzed by electrophoresis on a 2% agarose gel, and the voltage was applied at 150 V for 60 min. The electrophoretic results were recorded using the Gel Imaging System (Bio-Rad, USA).

### Stability of GQDs/Cy5-miR and GQDs/Cy5-miR@sEVs

The stabilities of GQDs/Cy5-miR and GQDs/Cy5-miR@sEVs were monitored in PBS (at room temperature) and in mouse serum (at 37 °C) for 50 min. GQDs/Cy5-miR and GQDs/Cy5-miR@sEVs (based on the equivalent dosage of 0.66 µg Cy5-miR) in respective volume of 100 µL were loaded in a 96-well black plate. Fluorescent measurements of Cy5-miR channel (λ_ex_/λ_em_ = 645/675 nm) were carried out using a microplate reader (Biotek, UK). 20 µg GQDs without Cy5-miR or sEVs were used as a control. The fluorescent signals were read once every 2 min, as the degradation rate (%) was calculated using the ratio of fluorescent intensity of the sample at each time point over that of 0.66 µg Cy5-miR. The stabilities of GQDs/FAM-miR and GQDs/FAM-miR@sEVs were monitored by the same way as above stated, except for the fluorescent channel of FAM-miR (λ_ex_/λ_em_ = 495/520 nm).

### Animal experiments

BALB/c nude mice (6 weeks old, body weight ~ 20 g) were purchased from the Cavens Laboratory Animal Co., Ltd (China). Mice were housed with free access to food and water in an ambient temperature-controlled room (23 ± 3 °C) with 12 h dark-light cycles and 40–70% humidity. All animal experiments were approved by the Institutional Animal Care and Use Committee (IACUC) of Jiangsu University. To establish mouse tumor models, 5.0 × 10^6^ HGC-27 cells were suspended in 200 µL PBS and injected subcutaneously onto the right rear flanks of each mouse. The mice bearing HGC-27 cells subcutaneous tumors were intravenously (i.v.) injected with Cy5-miR, GQDs/Cy5-miR and GQDs/Cy5-miR@sEVs (at an equivalent dose of 6.6 µg Cy5-miR/kg mouse body weight) and all injections were prepared in 200 µL via mouse tail vein. Then, real-time imaging was taken using In Vivo Imaging System (PerkinElmer, USA) at the Cy5 fluorescent channel (excitation at 640 nm and emission at 680 nm), following 24 h post injection (p.i.). Mice were sacrificed then, while the tumors and major organs were excised for ex vivo fluorescence imaging using the region-of-interest (ROI) analysis.

### In vivo therapeutic effects

To further visualize and evaluate tumor suppression of GQDs/Cy5-miR@sEVs, the tumor-bearing mice were i.v. injected with PBS, sEVs, Cy5-miR and GQDs/Cy5-miR@sEVs (at an equivalent dose of 6.6 µg Cy5-miR/kg mouse body weight, each with 200 µL injection) at 12, 15, 18, and 21 days after tumor inoculation. The real-time fluorescence imaging and ROI analysis of tumors were recorded after each injection. In each treatment, at 15 min, 1 h, 3 h, 6 and 12 h p.i., mice were examined at the Cy5 fluorescent channel. Tumor growth was monitored by digital calipers, and the volumes were calculated as $\text{Volume}=\frac{\text{Length}\times{(\text{Width})}^{2}}{{2}}$. Mouse body weights were also recorded during the treatment. After 22 d, the mice were sacrificed, and the tumors were excised and immediately weighted. Blood samples were collected to assess the liver function indexes of mice, including alanine aminotransferase (ALT) and aspartate aminotransferase (AST), and renal function indices typified by blood urea nitrogen (BUN) and creatinine (CREA). Dissection of collected organs and tumor tissues were processed using hematoxylin and eosin (H&E) staining, Ki-67 immunohistochemistry and (TUNEL) staining.

### Database

Targetscan Human 8.0 (http://www.targetscan.org), miRDB (http://www.mirdb.org) and PicTar (http://www.pictar.org) were used to analyze the target genes of selected miR-193a-3p, respectively. The Cancer Genome Atlas (TCGA) database (https://portal.gdc.cancer.gov/), Genotype-Tissue Expression Project (GTEx) database (https://www.genome.gov/Funded-Programs-Projects/Genotype-Tissue-Expression-Project), the Gene Expression Omnibus (GEO) database (https://www.ncbi.nlm.nih.gov/geo/) and the Kyoto Encyclopedia of Genes and Genomes (KEGG) database (https://www.genome.jp/kegg/) were used to explore the enrichment of the target genes of miR-193a-3p in GC.

## Results

### Preparation and identification of GQDs/Cy5-miR as a fluorescent switch

We first conducted predictive analysis on the target mRNA of miR-193a-3p. 29 genes were predicted to be the target of miR-193a-3p according to database tools, including PicTar, miRDB, TargetScan (Additional file 1: Fig. S1A). To screen this prediction, we then used TCGA and GTEx databases to compare the expression of all 29 miR-193a-3p-targeting mRNAs in GC tissues versus healthy tissues. The results indicated 18 genes among them are highly expressed in GC tissues (i.e., tumor expression level > normal tissue expression level, or T>N) (Additional file 1: Fig. S1B). Then RNA sequencing gene set (GSE77229) was obtained from GEO database. MiR-193a-3p mimics were transfected into SW900 cells (a cell line with deletions of the miR-193a locus) and the transcript abundance was analyzed by RNA sequencing. The downregulated transcripts were next analyzed for KEGG pathways enrichment. As a result, the repressed transcripts were mostly localized in the cell-cycle category and related genes were listed (Additional file 1: Table S1). The predicted target genes with T>N and the cell-cycle gene set were finally intersected to obtain 3-monooxygenase/tryptophan 5-monooxygenase activating protein zeta (*YWHAZ*) and cyclin D1 (*CCND1*) (Additional file 1: Fig. S1C). We next examined the gene expression of *YWHAZ* and *CCND1* in human GC cells lines including HGC-27, MKN-28, AGS and SNU-1, and the normal human gastric mucosal epithelial cell line (GES-1) by qRT-PCR. The results displayed the high gene expression of both *YWHAZ* and *CCND1* in the HGC-27 cells line, where *CCND1* was most significantly expressed (Additional file 1: Fig. S1D). The combination of miR-193a-3p and its target gene *CCND1* was based on the complementary base pairing in seed region (red) (Additional file 1: Fig. S1E). Therefore, through this examination, we selected *CCND1* as the target gene of miR-193a-3p in the HGC-27 cells.

We next fabricated the Cy5/miR nanocomplex for miRNA delivery. Firstly, the size of commercially available GQDs was analyzed by high-resolution transmission electron microscopy (HR-TEM). The results showed that the diameter of GQDs was 5 nm, and the lattice fringe was 0.21 nm (Additional file 1: Fig. S2A). The absorbance spectra of GQDs reached a peak at ~ 340 nm (Additional file 1: Fig. S2B) and upon excitation at 360 nm (determined by excitation spectra as the maximal excitation wavelength), the emission peak occurred at 450 nm (Additional file 1: Fig. S2C), in line with the photoluminescent properties of GQDs. Secondly, miR-193a-3p was chemically tagged with either FAM or Cy5 fluorescent dyes. The fluorescent intensity of GQDs (λ_ex_/λ_em_ = 360/450 nm), Cy5-miR (λ_ex_/λ_em_ = 645/675 nm) or FAM-miR (λ_ex_/λ_em_ = 495/520 nm) was individually measured in a series of working solutions, validating the linear range of their fluorescent emission intensity over concentrations (Additional file 1: Fig. S2D–F).

**Fig. 2:**
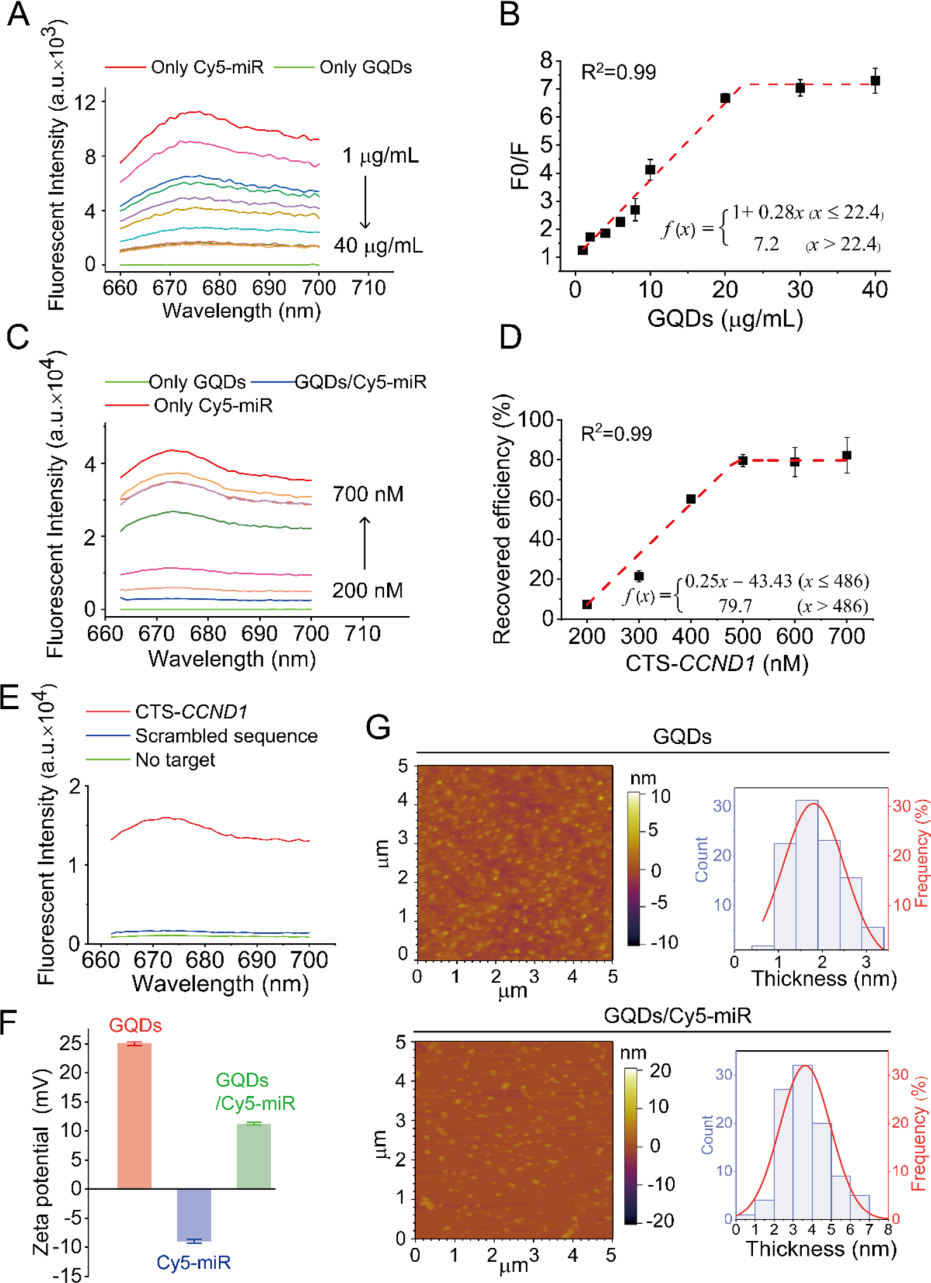
The interactions between the Cy5-tagged miRNAs and GQDs, together with physicochemical properties of the formed nanocomplexes. **A** Fluorescent intensity of Cy5-miR (100 nM) in the presence of varying concentrations of GQDs (1, 2, 4, 6, 8, 10, 20, 30 and 40 µg/mL). **B** The fluorescent quenching efficiency of Cy5-miR (100 nM) and GQDs was calculated. *F* Fluorescent intensity of the Cy5-miR interacted with varying concentrations of GQDs, *F0* fluorescent intensity of Cy5-miR without GQDs. **C** Fluorescent intensity of GQDs/Cy5-miR in the solution after incubating with different concentrations of CTS-*CCND1* (200, 300, 400, 500, 600 and 700 nM). **D** Fluorescent recovery efficiency calculated to determine the minimum ratio of CTS-*CCND1* for miR-193a-3p release in GQDs/Cy5-miR. **E** Fluorescence intensity of GQDs/Cy5-miR under different conditions: with no target, CTS-*CCND1* or scramble sequence. **F** Zeta potentials of GQDs and GQDs/Cy5-miR were measured. **G** AFM images and topographic height histograms of GQDs and GQDs/Cy5-miR

We then evaluated the fluorescence quenching of Cy5-miR in the presence of various concentrations of GQDs at 37 °C. As the aromatic moiety of Cy5 fluorescent tag forms π–π stacking with the planar graphene sheet, the decreasing fluorescence of Cy5 was observed when the constant Cy5-miR was incubated with increasing GQDs in the Tris-HCl buffer (Fig. 2A). As a result, a maximum of (85.0 ± 0.4)% Cy5 fluorescence was quenched when adequate GQDs was added. Thus, the GQDs/Cy5-miR nanocomplexes were prepared with an optimized mass ratio of GQDs:Cy5-miR = 30:1 (Fig. 2B). Then, the complementary target sequence in *CCND1* (CTS-*CCND1*) was added into the solutions. Resultantly, the Cy5 fluorescence was gradually recovered with the increasing concentration of CTS-*CCND1*, and a final recovery of (82.4 ± 4.1)% Cy5 fluorescence (the ratio of the original Cy5 fluorescence) was attained (Fig. 2C). The optimized molar ratio of required CTS-*CCND1* over Cy5-miR was 6:1 (Fig. 2D). By switching the fluorescent moiety from Cy5 to FAM, the fluorescence of FAM-miR showed a resemblance of quenching when adding GQDs due to the increasing π–π stacking and the minimized fluorescence was detected when the mass ratio of GQDs: FAM-miR = 100:1 (Additional file 1: Fig. S3A, B). Similarly, FAM fluorescence was gradually recovered when CTS-*CCND1* was further added, and this fluorescence remained unchanged as the molar ratio of CTS-*CCND1* over FAM-miR reached 6:1 and above (Additional file 1: Fig. S3C, D).

To investigate whether GQDs/Cy5-miR or GQDs/FAM-miR can bind CTS-*CCND1* specifically, a noncomplementary oligonucleotide strand with random nucleotide sequence was synthesized and described as scramble sequence. GQDs/Cy5-miR or GQDs/FAM-miR were incubated in aqueous solutions with scramble sequence or CTS-*CCND1* to confirm their sequence specificity. The Cy5 fluorescence of GQDs/Cy5-miR was enhanced 14.7-folds after incubation with CTS-*CCND1*, while the fluorescence went through minor change in the presence of scramble RNA. This result suggested that Cy5-miR in the GQDs/Cy5-miR complex failed to bind the scramble sequence, but it recognized and bound CTS-*CCND1* with high specificity (Fig. 2E). The similar experiments using GQDs/FAM-miR were also performed, showing its specific recognition and binding of CTS-*CCND1* (Additional file 1: Fig. S4). In addition, zeta potentials of all materials in solutions (pH = 7.5) were examined with results shown in Fig. 2F. GQDs demonstrated a positive surface charge of (25.0 ± 0.3) mV, and Cy5-miR showed a negative charge of (− 9.0 ± 0.3) mV, while the particles of GQDs/Cy5-miR owned an overall surface charge of (11.3 ± 0.2) mV. Concurrently, the morphology of GQDs and GQDs/Cy5-miR nanocomplex was examined by atomic force microscope (AFM). GQDs exhibited a typical nano-sized lamella structure with an average height of ~ 2 nm. In contrast, GQDs/Cy5-miR had distinctly increased thickness of ~ 4 nm (Fig. 2G).

### Fabrication, characterization and stability of GQDs/Cy5-miR@sEVs

Next, we sought to encapsulate GQDs/Cy5-miR within sEVs to fabricate the dual-functional nanoplatform to induce and visualize their antitumor effects. We first isolated hucMSCs from healthy donors and verified their biological characteristics. Using flow cytometric analysis, we confirmed that the obtained MSCs expressed CD90, CD105, CD73, and CD44, but not CD34 or CD45 (Additional file 1: Fig. S5A). Further analyses using Alizarin Red and Oil Red O staining indicated that these MSCs demonstrated osteogenic and adipogenic differentiation capabilities (Additional file 1: Fig. S5B).

Following the isolation and purification of hucMSCs-derived sEVs, we then optimized the technical parameters to load GQDs/Cy5-miR into sEVs via sonication. Firstly, we examined the morphological structure of sEVs by transmission electron microscopy (TEM) and measured their particle size distribution detected by nanoparticle tracking analysis (NTA) after sEVs were sonicated under various output powers. TEM images showed that the lipid bilayer membrane of sEVs treated with 320 joules sonication output power was damaged, further confirmed by NTA results suggestive of membrane structure cracks in sEVs. By lowering the sonication output power to 250 joules, the treated sEVs remained intact as observed by TEM, and their particle size (peaked at ~ 150 nm) showed no significant difference from untreated sEVs (Additional file 1: Fig. S6A, B). Therefore, we chose 250 joules as the suitable sonication output power to load GQDs/Cy5-miR into sEVs.

**Fig. 3:**
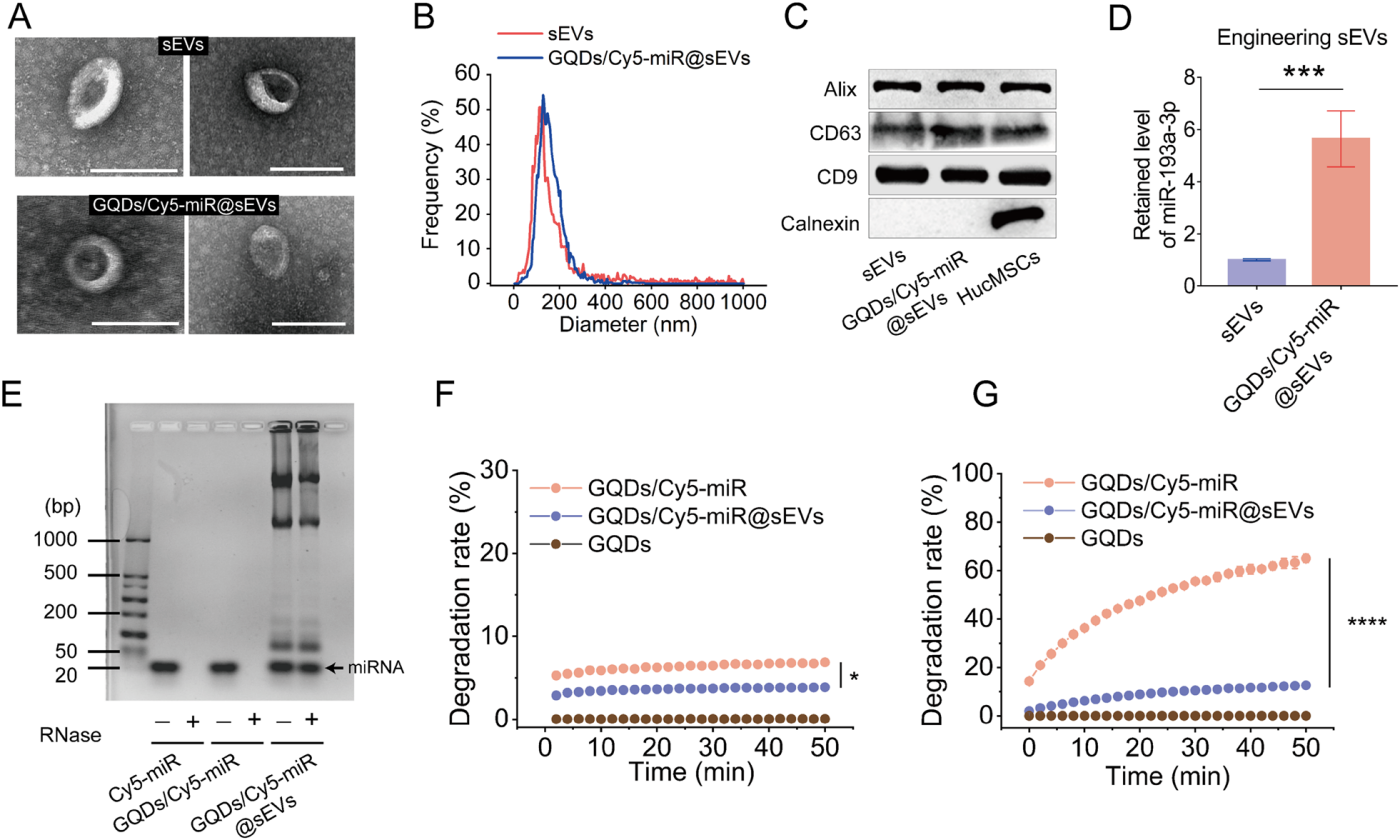
Characterization and drug loading capacity of GQDs/Cy5-miR@sEVs. **A** TEM images of sEVs (upper panel) and GQDs/Cy5-miR@sEVs (lower panel). Scale bar = 200 nm. **B** Size distribution of sEVs and GQDs/Cy5-miR@sEVs. **C** Expression of Alix, CD63, CD9, Calnexin in sEVs, GQDs/Cy5-miR@sEVs and HucMSCs was detected by Western blot. **D** MiR-193a-3p in GQDs/Cy5-miR@sEVs and sEVs were detected by qRT-PCR. **E** The degradation of Cy5-miR, GQDs/Cy5-miR and GQDs/Cy5-miR@sEVs in RNase solutions was detected by agarose gel electrophoresis. **F** Stability of GQDs/Cy5-miR and GQDs/Cy5-miR@sEVs in PBS in 50 min at room temperature. **G** Stability of GQDs/Cy5-miR and GQDs/Cy5-miR@sEVs in 50 min after being incubated within mouse serum at 37 °C. Data are expressed as mean ± SD and analyzed by one-way ANOVA. *p < 0.05, ***p < 0.001, ****p < 0.0001

Concurrently, the morphological structures of the untreated sEVs and GQDs/Cy5-miR@sEVs were revealed by TEM images (Fig. 3A). The particle sizes of untreated sEVs and GQDs/Cy5-miR@sEVs were determined to be 139.1 ± 19.0 nm and 149.7 ± 3.7 nm (mean ± SD) by NTA, respectively (Fig. 3B). The Western blotting results confirmed the expression of positive biomarker CD9, CD63, and Alix in sEVs, GQDs/Cy5-miR@sEVs and hucMSCs, with calnexin absent in sEVs and GQDs/Cy5-miR@sEVs as the negative biomarker (Fig. 3C). The miRNA contents of GQDs/Cy5-miR@sEVs were further analyzed using qRT-PCR to calculate the drug loading capacity (LC = the ratio of EV-encapsulated amount to the initial amount of cargo). The results showed that the loading capacity for miR-193a-3p was 5.6 times higher in GQDs/Cy5-miR@sEVs than the untreated sEVs (Fig. 3D), which corroborated the substantial inclusion of exogenous miR-193a-3p into GQDs/Cy5-miR@sEVs.

We then evaluated the stability of miRNA in the forms of Cy5-miR, GQDs/Cy5-miR, and GQDs/Cy5-miR@sEVs after RNases treatment. Agarose gel electrophoresis experiments displayed that miRNA contents in Cy5-miR and GQDs/Cy5-miR were degraded by RNases. For the condition containing GQDs/Cy5-miR@sEVs, miRNA band was observed, indicating that the miRNA could be largely protected from RNase degradation once encapsulated inside GQDs/Cy5-miR@sEVs (Fig. 3E).

To assess the stability of the GQDs/Cy5-miR@sEVs in PBS, GQDs/Cy5-miR and GQDs/Cy5-miR@sEVs were added to PBS for ~ 1 h and degradation kinetics of Cy5-miR from GQDs/Cy5-miR@sEVs were evaluated by fluorescence detection of Cy5-miR. As a result, 3.9% fluorescence of GQDs/Cy5-miR@sEVs decayed in 50 min, while 6.9% fluorescence of GQDs/Cy5-miR went off during the same time (Fig. 3F). Next, GQDs/Cy5-miR and GQDs/Cy5-miR@sEVs were added to mouse serum and incubated at 37 °C for ~ 1 h. The stability of GQDs/Cy5-miR and GQDs/Cy5-miR@sEVs in mouse serum was assessed by monitoring Cy5 fluorescence tracer. The results showed that 65.1% fluorescence of GQDs/Cy5-miR decayed in serum while only 12.6% fluorescence of GQDs/Cy5-miR@sEVs vanished, suggesting a higher stability of fluorescence switch when included in sEVs (Fig. 3G). This result confirmed that sEVs effectively protected the encapsulated miRNA from RNase degradation, especially when exposed to physiological milieu.

### Verifying the fluorescence switching and tumor inhibition of GQDs/Cy5-miR@sEVs in vitro

**Fig. 4:**
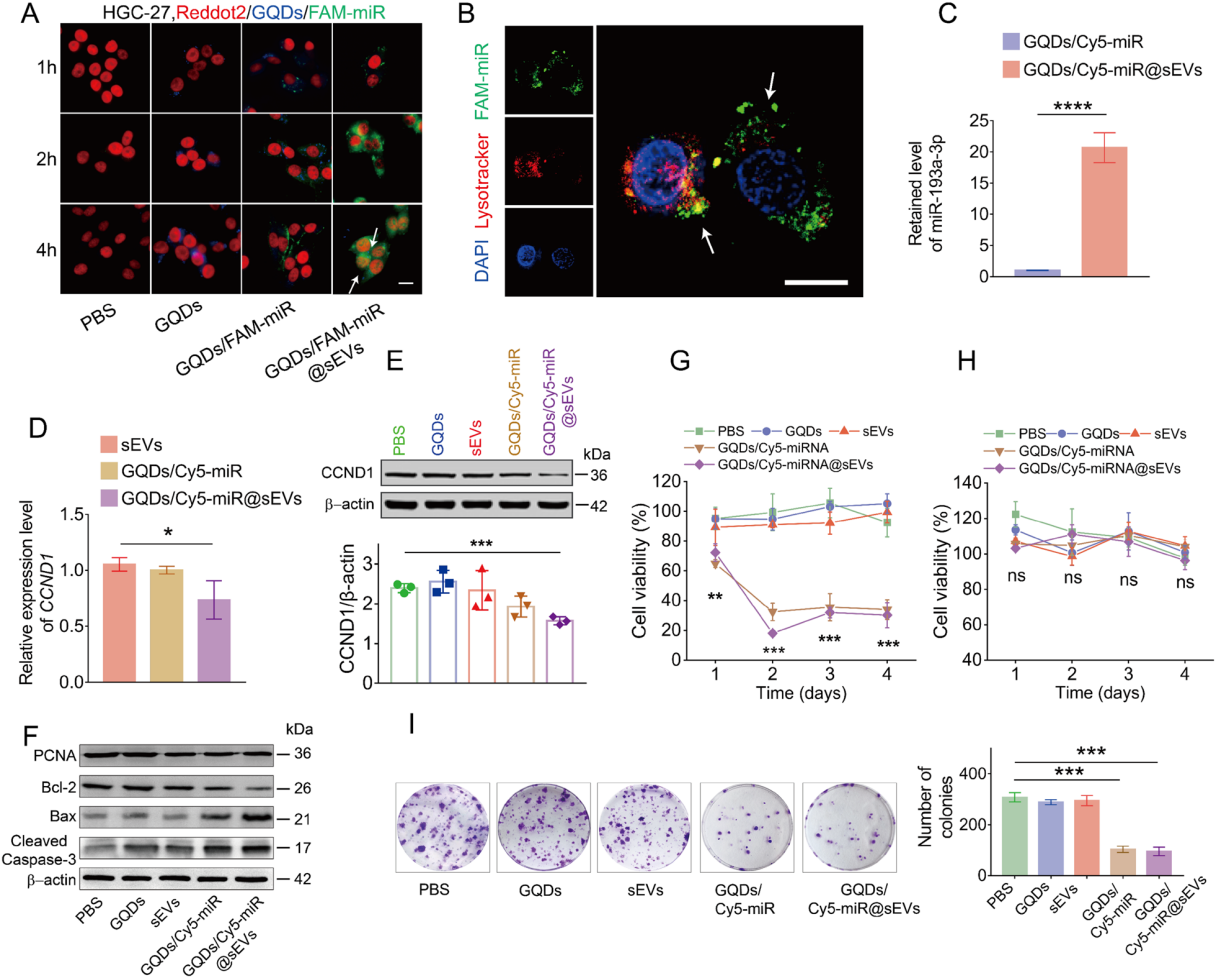
In vitro fluorescent “off–on” switch and antitumor activity of GQDs/Cy5-miR@sEVs. **A** The fluorescence images showing the uptake of FAM-miR-GQDs@sEVs by HGC-27 cells at 1, 2, and 4 h. GQDs (blue), FAM-tagged miRNA (green) and nuclei (red) were shown, where scale bar = 100 μm. **B** FAM-miRNA (green) successfully escaped from endosomes (red) as evidenced by the separation of green and red fluorescence (indicated by arrows). Endosome/lysosome and nuclei were stained with Lysotracker Red and DAPI, respectively. Scale bar = 20 μm. **C** MiR-193a-3p was detected in HGC-27 cells by qRT-PCR after they were treated with GQDs/Cy5-miR and GQDs/Cy5-miR@sEVs. **D** Target gene *CCND1* was detected in HGC-27 cells by qRT-PCR after they were treated with sEVs, GQDs/Cy5-miR and GQDs/Cy5-miR@sEVs, respectively. **E** Protein expression of CCND1 in HGC-27 cells by Western blot after being treated with PBS, GQDs, sEVs, GQDs/Cy5-miR, and GQDs/Cy5-miR@sEVs, respectively. **F** Western blot was used to detect the protein level of PCNA, Bax, Bcl-2, cleaved caspase-3 as well as β-actin proteins in HGC-27 cells. **G** Viability of HGC-27 cells and **H** viability of HUVEC cells were determined by CCK-8 assay. **I** Cell colony formation assays of HGC-27 cells when treated in different conditions as indicated. Data are expressed as mean ± SD and analyzed by one-way ANOVA. ns, no significance; *p < 0.05, **p < 0.01, ***p < 0.001, ****p < 0.0001

Following the fabrication and characterization of GQDs/Cy5-miR@sEVs, we investigated the cellular uptake and “off–on” fluorescence switch of those particles. To assess miRNA delivery and gene regulation of GQDs/FAM-miR@sEVs in vitro, GQDs, GQDs/FAM-miR and GQDs/FAM-miR@sEVs were individually incubated with HGC-27 cells for 1, 2 and 4 h, respectively. Notably, we here adopt GQDs/FAM-miR@sEVs instead of GQDs/Cy5-miR@sEVs because reddot2 dye is used in this experiment to label the nuclei, which concomitantly overlaps with Cy5 fluorescent channel. As a result, images taken by confocal laser scanning microscope (CLSM) showed that HGC-27 cells treated by GQDs/FAM-miR@sEVs exhibited high FAM-miR uptake levels and successful fluorescent “off–on” switch, demonstrated by the observed FAM fluorescence in cells at 2 h that continued to glow inside cytoplasm and nucleus up to 4 h. This observation verified the FAM-miR detached from GQDs and bound to its target genes *CCND1* in living cells. Oppositely, the FAM fluorescence in GQDs/FAM-miR treatment remained nearly undetectable in 4 h, suggesting the bindings between miR-193a-3p and its target genes *CCND1* rarely occurred on this condition (Fig. 4A).

This was supported by subcellular CCND1 mRNA localization studies utilizing RNA fluorescence in situ hybridization (RNA-FISH) technique. CLSM images showed that *CCND1* mRNA was distributed in both nuclei and cytoplasm of HGC cells, explaining the spatial “off–on” switch of fluorescence during GQDs/FAM-miR trafficking inside cells (Additional file 1: Fig. S7). These results demonstrated that the sEVs encapsulation assisted the fluorescence switch GQDs/FAM-miR to relocate into cells. In order to track the released miR-193-3p from GQDs/FAM-miR@sEVs following cell internalization, the intracellular localization of FAM-miR was further examined after GQDs/FAM-miR@sEVs were incubated with HGC-27 cells for 2 h. As shown in Fig. 4B, FAM-miR in the GQDs/FAM-miR@sEVs was able to escape from the endosome/lysosome trapping, as evidenced by minimal overlapping of green (FAM-miR) and red (Lyso-tracker Red) fluorescence in the cytoplasm.

Then, the miR-193a-3p miRNA and its target gene *CCND1* were detected by qRT-PCR after HGC-27 cells were treated with GQDs/Cy5-miR@sEVs or GQDs/Cy5-miR. The relative expression of miR-193a-3p in GQDs/Cy5-miR@sEVs showed an increase of 20.7-folds when compared with that in GQDs/Cy5-miR (Fig. 4C). Next, the gene expression of *CCND1* exhibited a significant decline (> 20%) in GQDs/Cy5-miR@sEVs when compared with that in GQDs/Cy5-miR or in sEVs (Fig. 4D). Taken together, our results validated that GQDs/Cy5-miR@sEVs could effectively deliver miR-193a-3p into HGC-27 cells and substantially downregulate the transcription of its target gene *CCND1*.

Simultaneously, the Western blotting analysis suggested that the expression of CCND1 protein in HGC-27 cells declined after being treated with GQDs/Cy5-miR@sEVs for 48 h, while the cells treatment with GQDs, sEVs or GQDs/Cy5-miR revealed no noticeable changes in the CCND1 protein expression (Fig. 4E). CCND1 protein is well known as a key regulator in arresting cell cycle and inducing cell apoptosis. Consequently, we monitored the apoptosis markers in HGC-27 cells treated with GQDs/Cy5-miR@sEVs and other indicated groups. GQDs/Cy5-miR@sEVs significantly decreased the proliferating cell nuclear antigen (PCNA) protein expression level in HGC-27 cells. The level of the anti-apoptosis protein B-cell lymphoma-2 (Bcl-2) was significantly reduced, whilst the pro-apoptosis protein Bcl-2 associated X (Bax) and cleaved caspase 3 were increased (Fig. 4F).

Cell viability tests showed that the viability of GQDs/Cy5-miR and GQDs/Cy5-miR@sEVs treated cells deceased to (32.4 ± 5.9) % and (18.1 ± 1.7) % (*p* < 0.001) after 48 h, respectively, whereas the treatment with GQDs or sEVs showed no statistical difference from the untreated (Fig. 4G). The same experiments were further performed on HUVEC cells, and the results revealed that GQDs/Cy5-miR@sEVs had no adverse effect on non-cancerous HUVEC cells (Fig. 4H).

To further assess the growth inhibition of tumor cells in vitro, HGC-27 cells were treated with different concentrations of GQDs/Cy5-miR@sEVs from 10 to 200 µg/mL (based on the total protein mass concentration of sEVs). Results revealed that GQDs/Cy5-miR@sEVs suppressed the tumor cell growth in a dose-dependent manner at 48 h when its concentration was within 100 µg/mL (Additional file 1: Fig. S9). Moreover, the cell colony formation assay demonstrated that GQDs/Cy5-miR and GQDs/Cy5-miR@sEVs induced the fewest colony formations and exhibited the most durable therapeutic effect among all groups (Fig. 4I). Transwell migration and invasion tests also revealed that GQDs/Cy5-miR and GQDs/Cy5-miR@sEVs most significantly prevented the migration and invasion of HGC-27 cells among all conditions (Additional file 1: Fig. S10). These results verified that both GQDs/Cy5-miR and GQDs/Cy5-miR@sEVs could promote miRNA delivery to inhibit the growth and proliferation of HGC-27 cells in vitro.

### The anticancer effect of GQDs/Cy5-miR@sEVs in tumor-bearing mice visualized by their fluorescence switch

**Fig. 5:**
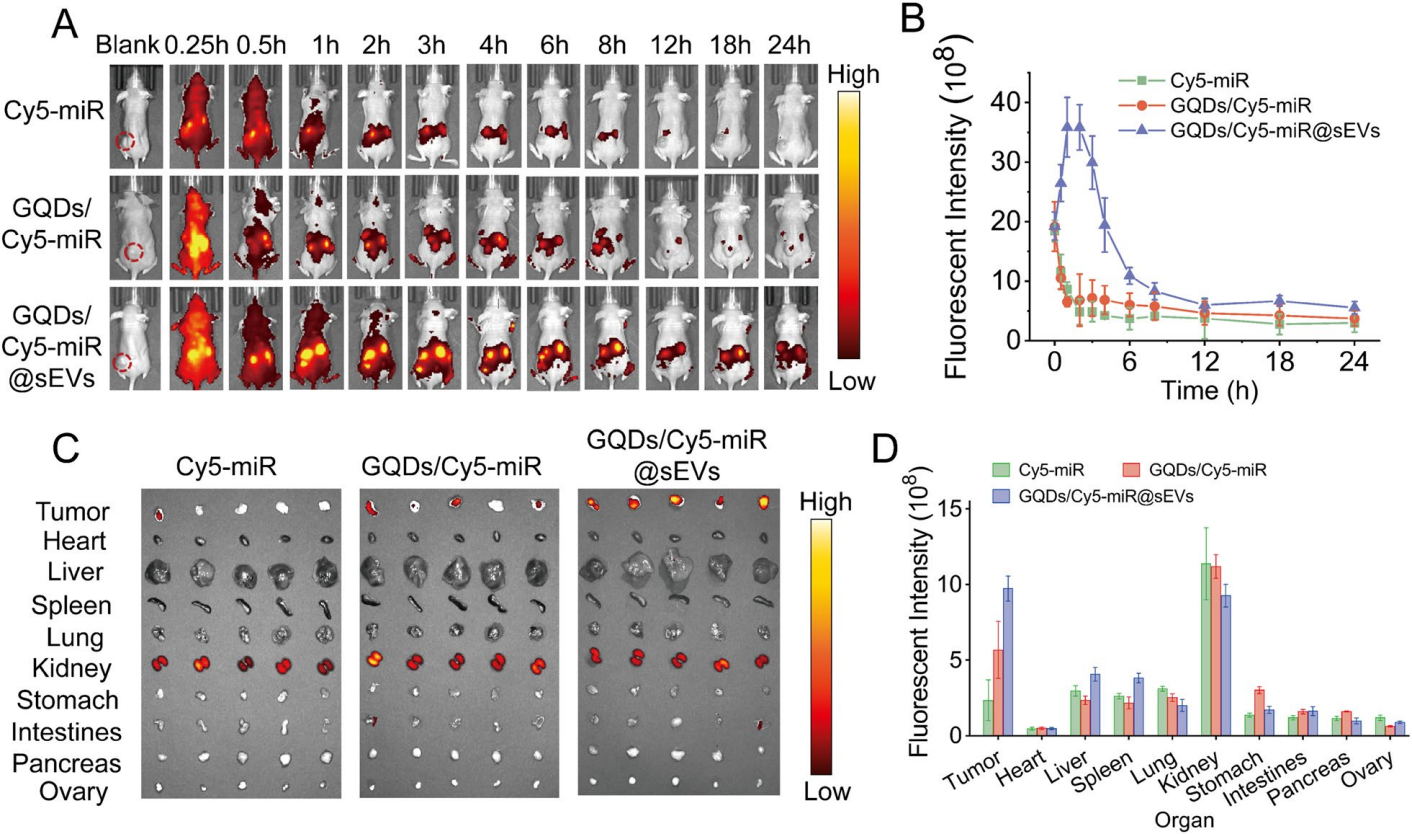
In vivo fluorescent “off–on” switch effect of GQDs/Cy5-miR@sEVs. **A** The fluorescent images of tumor-bearing mice after i.v. injection of GQDs/Cy5-miR@sEVs at time points as indicated. **B** The fluorescent intensity of tumor sites in vivo at indicated time points. **C** Ex vivo fluorescent signals in major organs (i.e., liver, spleen, lung, heart, kidney, stomach, intestines, pancreas, and ovary) and tumors 24 h p.i. **D** Ex vivo fluorescent intensity of major organs and tumors 24 h p.i

We next evaluated the fluorescence switching effect of GQDs/Cy5-miR@sEVs in a xenograft murine tumor model established by subcutaneous injection of HGC-27 cells in the flanks of BALB/c nude mice. Each group (n = 5) of mice was i.v. injected through tail vein with Cy5-miR, GQDs/Cy5-miR, and GQDs/Cy5-miR@sEVs, respectively (based on the equivalent dosage of Cy5-miR). Tumor accumulation and biodistribution of the Cy5-miR were monitored within 24 h p.i. As a result, attenuated fluorescence signals were detected at the tumor sites for the GQDs/Cy5-miR or Cy5-miR treated mice. In contrast, much higher fluorescence signals in tumors of mice injected with GQDs/Cy5-miR@sEVs were noticed, reaching a peak at 1 h p.i. and sustaining up to 6 h p.i. (Fig. 5A, B). After 24 h p.i., the mice were sacrificed before the major organs and tumors were harvested to examine the fluorescence biodistribution. As shown in Fig. 5C, D, the Cy5 fluorescence was mainly accumulated in the kidneys and tumors for each substance administered. In comparison, GQDs/Cy5-miR@sEVs showed higher accumulation than Cy5-miR or GQDs/Cy5-miR in tumors.

**Fig. 6:**
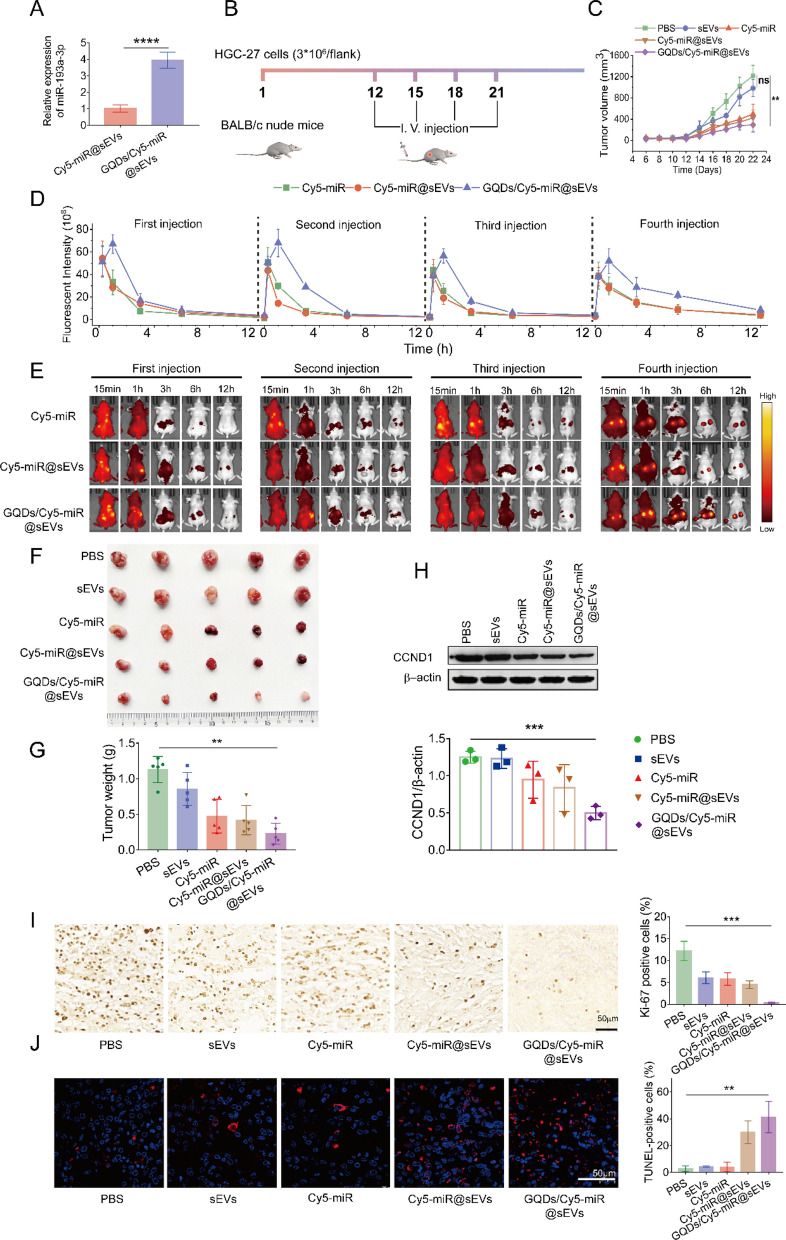
Visualized treatment of GQDs/Cy5-miR@sEVs in the tumor-bearing murine model. **A** The miR-193a-3p in GQDs/Cy5-miR@sEVs and Cy5-miR@sEVs was detected by qRT-PCR. **B** Scheme illustrating GQDs/Cy5-miR@sEVs treatment in subcutaneous xenograft tumor model established by HGC-27 cells in BALB/c nude mice. **C** The in vivo tumor volumes were monitored over time during four injection cycles. **D**, **E** The fluorescence intensity of tumor region was monitored and plotted over time. **F** Images of subcutaneous xenograft tumors in mice in each group (n = 5) when the tumor sizes were measured ex vivo. **G** Tumor weights of mice in each group were measured. **H** Western blot assays detected the expression of CCND1 protein in different treatments. **I** Ki-67 and **J** TUNEL staining of tumors from mice in different groups. Scale bars = 50 μm. One-way ANOVA for multiple groups was applied for statistical analysis. ns, no significance; **p < 0.01 and ***p < 0.001

We further evaluated the therapeutic effect of GQDs/Cy5-miR@sEVs on the subcutaneous tumor model established by xenograft HGC-27 cells injection in BALB/c nude mice. Notably, as miRNA was the therapeutic segment in GQDs/Cy5-miR@sEVs, we here included Cy5-miR@sEVs for a comparison. The fabrication of Cy5-miR@sEVs followed the same procedure as preparing GQDs/Cy5-miR@sEVs, using the equivalent amount of Cy5-miR. Then, the miRNA loading capacity of Cy5-miR@sEVs and GQDs/Cy5-miR@sEVs was compared. Results indicated that the content of miRNA in GQDs/Cy5-miR@sEVs was nearly fourfold of that in Cy5-miR@sEVs based on the same total protein mass of sEVs (Fig. 6A).

Then, each group of mice (n = 5) was i.v. administered with PBS, sEVs, Cy5-miR, and Cy5-miR@sEVs, and GQDs/Cy5-miR@sEVs (based on the equivalent dosage of Cy5-miR) for 4 cycles (3 days per cycle) (Fig. 6B) and the tumors growth in mice was monitored (Fig. 6C). During a total of 4 injections, individually at 12, 15, 18, and 21 days after tumor inoculation, the fluorescent intensities of tumors were monitored following 24 h p.i. (Fig. 6D, E). Substantial tumor growth was detected in PBS group, while mice with Cy5-miR, sEVs, or GQDs/Cy5-miR treatment revealed moderate tumor suppression. In contrast, the tumor growth in mice treated with GQDs/Cy5-miR@sEVs was significantly inhibited (Fig. 6F, G).

**Fig. 7:**
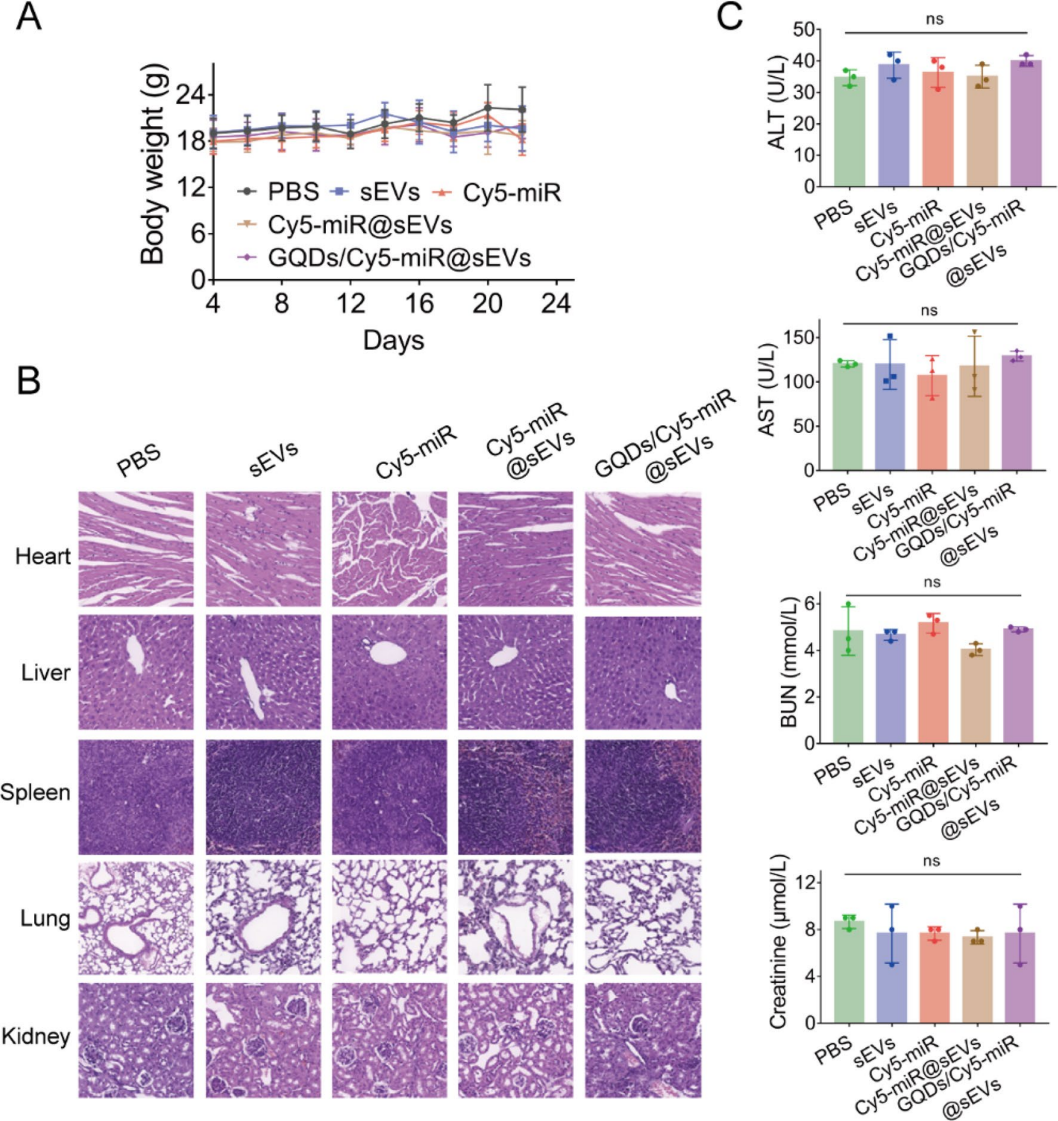
The biosafety evaluation of GQDs/Cy5-miR@sEVs in vivo. BALB/c nude mice were sacrificed after administration with PBS, sEVs, Cy5-miR@sEVs and GQDs/Cy5-miR@sEVs. **A** Body weights of mice subject to different treatments as indicated. **B** The major organs of mice in each group including liver, spleen, heart, lung, and kidney were collected for H&E staining. **C** The measured levels of biochemical indicators in the mouse blood samples, including the indices for liver (ALT and AST) and kidney (BUN and CREA). Data are expressed as mean ± SD and analyzed by one-way ANOVA. ns, no significance

Concomitantly, the Western blotting assays demonstrated a distinctive decrease of CCND1 expression in mice treated with GQDs/Cy5-miR@sEVs compared to any of other treatments (Fig. 6H). Furthermore, the number of Ki-67 positive cells was significantly decreased, and the number of terminal deoxynucleotidyl transferase-mediated dUTP-biotin nick end labeling (TUNEL) positive cells was notably increased after treatment with GQDs/Cy5-miR@sEVs, in comparison to that under other condition (Fig. 6I, J). These results confirmed that GQDs/Cy5-miR@sEVs could effectively induce cell apoptosis in tumors, showing their prominent capability on tumor inhibition in vivo. Simultaneously, for GQDs/Cy5-miR@sEVs treatment, there was no significant change in mouse body weight (Fig. 7A), nor pathological changes in main organs (Fig. 7B), nor any abnormality noticed in major hepatic and renal functions (Fig. 7C). These results reflected that GQDs/Cy5-miR@sEVs possessed a tumor-specific toxicity while bearing a very good biocompatibility.

## Discussion

In this study we obtained hucMSCs from the healthy donors and purified the hucMSC-derived sEVs, followed by the encapsulation of GQDs/Cy5-miR fluorescent nanocomplexes into sEVs via sonication. The sEVs-encapsulated GQDs/Cy5-miR exhibited efficient cellular uptake and endosomal escape, and significant tumor accumulation and inhibition due to the enhanced delivery of functional miR-193a-3p that binds to its target gene *CCND1*. Simultaneously, the fluorescent tag in GQDs/Cy5-miR enabled in vivo visualization of tumor therapeutics as the binding between miR-193a-3p and its target gene *CCND1* occurred. Our results here demonstrated the capability of hucMSC-derived sEVs as an excellent delivery nanoplatform for exogenous nucleic acids, and as a valid tool for efficient visualization of pharmaceutical miRNA in vivo.

Several drug loading methods into sEVs prevail in current biomedical research, typified by parent cell manipulation, sonication, and electroporation, albeit the cargo encapsulation efficiency is far from satisfying. Among those protocols, parent cell treatment suits only for preparation of the cell line derived sEVs rather than EVs from body fluids [10, 26]. In parallel, electroporation could induce the RNA aggregation, further lowering the loading capacity in the drug delivery [27]. Presently, facile and efficient cargo encapsulation remains a challenging prerequisite for wide applications of sEVs in the pharmaceutical delivery regimen.

Alternatively, sonication has been suggested as an optimized sEVs loading method with a comparatively high efficiency [26, 28]. Most previously reported sonication methods were mainly used for cell drug loading and bioengineering operation, which could generate pores ranged from 100 nm to 1.25 μm on the cell membrane [29]. Concurrently, sonication could bring a sudden energy burst that may undermine the membrane structure of sEVs and deactivate proteins on the outer surface. Given those risks, sonicated parameters previously applied may not be suitable for sEVs (particle size = ~ 30–200 nm) manipulation. To overcome the drawbacks of traditional sonication, our study here optimized tunable sonication and tested different parameters to find a reproducible condition for efficient sEVs loading. Energy output and working frequency were quantified and adjusted to secure a good loading efficiency while keeping sEVs membrane intact. As a result, the loading efficiency of miR-193a-3p that was loaded into sEVs in the form of GQDs/Cy5-miR via sonication was increased by 5.6-fold of that in the unsonicated group, superior to other studies using miRNA loading methods via sonication (2.7-fold) [28], electroporation (> 3-fold) [30], and drug stimulation (~ 4-fold) [31].

In the past decades, the study of miRNA delivery in vivo focuses on liposomes [32], cationic polymers [33], and hydrogel microspheres [34]. EVs-based delivery platform has recently attracted considerable research attention. The advantages of sEVs as a potent miRNA delivery system include their low immunogenicity [35], suitable mechanical properties [36], and inherent targeting ability (from their parent cells) [37, 38]. Compared to synthetic particles, of which up to 90% of the injection amount ends up in the liver, sEVs might minimize their clearance by reticuloendothelial system down to 23% [17, 39]. Intriguingly, hucMSCs-derived sEVs could stay in tumors ~ 36 h longer than artificial nanoparticles of comparable sizes [40]. However, for most delivery systems, it is challenging to accurately track the distribution and long-term fate of encapsulated pharmaceutical cargoes even equipped with traceable tags.

To monitor these delivery, several noninvasive methods have been long applied, including positron emission tomography/computed tomography [41], mass spectrometry [42], and fluorescence imaging that emits at visible (400–700 nm), near-infrared (NIR)-I (700–900 nm), or NIR-II (1000–1700 nm) window [43, 44]. Fluorescence imaging methods have the advantages of high imaging resolution, easy operating procedure, and low economical cost, which are conducive to multichannel fluorescence monitoring of drug delivery systems in cells and animals. On this aspect, a graphene-based fluorescent nanoprobe was designed to combine both drug visualization and cargo-loading into one vehicle, using layered double hydroxides (LDHs) for cancer targeting and etoposide (VP16) for chemotherapy, both seated in sulfur-doped graphene quantum dots (SGQDs) [45]. These LDH@SGQD-VP16 particles could effectively accumulate in the tumor microenvironment while monitored by real-time fluorescence imaging.

Towards advancing this theranostic combination concept, we here fabricated GQDs/Cy5-miRNA@sEVs as a promising strategy to visualize the release of the delivered miR-193a-3p both in vitro and in vivo with a visible fluorescent signal switch and to downregulate the *CCDN1*, a typically oncogenic gene in GC [46]. In particular, the sEVs-delivered fluorescent (FAM and Cy5) probes showed the great specificity for target gene *CCND1*, high serum stability and excellent endosomal escape capacity. Benefiting from these intrinsic characteristics and functions, GQDs/Cy5-miR@sEVs presented unmatched advantages in miRNA delivery in vivo over GQDs/Cy5-miR, although they exhibited similar tumor cell inhibition in vitro. This makes our EV approach a superior choice for oligonucleotide drug delivery.

## Conclusion

In this study we fabricated GQDs/Cy5-miRNA@sEVs nanoparticles that could be visualized for their cancer therapeutic efficiency through off-to-on switch of the fluorescence signal as the enclosed GQDs/Cy5-miRNA combined with its targeted mRNA in cancerous cells and in tumor-bearing mice. This work provides a useful tool to assess and visualize the delivery efficiency of pharmaceutical nucleic acids in vitro and in vivo.

### Supplementary Information

**Additional file 1: Table S1.** Top 3 enriched Kyoto Encyclopedia of Genes and Genomes (KEGG) pathways for genes that were significantly downregulated by miR-193a-3p [1]. **Figure S1.** Identification of *CCND1* as a target gene for miR-193a-3p delivery. (A) Venn diagram of miR-193a-3p target genes in three databases (PicTar, miRDB, TargetScan). (B) The expression of 29 target genes in tumor tissues and normal tissues according to TCGA and GTEx databases. The 18 underscored genes show higher expression in GC tissues than in normal tissues (T>N). (C) Venn diagram of two gene sets. Predicted target genes (T>N), a gene set that was upregulated in GC tissues identified by TCGA and GTEx databases. Cell-cycle category, a gene set that was maximally regulated by miR-193a-3p from GEO database. (D) qRT-PCR assays showing the mRNA expression of *YWHAZ* and *CCND1* in GC cell lines (HGC-27, MKN-28, AGS, SNU-1) and a normal human gastric mucosal epithelial cell line (GES-1). (E) The combination of miR-193a-3p and its target gene *CCND1*. **Figure S2.** Preparation and characterization of GQDs and miR-193a-3p. (A) HR-TEM images of GQDs. (B) UV–Vis absorption of GQDs from 200 to 800 nm. (C) Emissions spectra of GQDs when excited at various wavelengths in dilute aqueous solutions. (D) Fluorescent intensity of GQDs in the concentration of 2, 5, 10, 20, 30, 40, and 50 μg/mL, respectively. (E) Fluorescent intensity of Cy5-miR in the concentration of 20, 50, 100, 200, 300, 400, and 500 nM, respectively. (F) Fluorescent intensity of FAM-miR with the concentration of 20, 50, 100, 200, 350, and 500 nM, respectively. **Figure S3.** The interactions between the FAM-tagged miRNAs and GQDs. (A) Fluorescent intensity of FAM-miR (100 nM) in the presence of varying concentrations of GQDs (2, 6, 8, 10, 20, 30, 40, 50, 60, 70 and 80 μg/mL) (B) The fluorescent quenching efficiency of FAM-miR (100 nM) and GQDs was calculated. F0: Fluorescent intensity of FAM-miR without GQDs. F: Fluorescent intensity of the FAM-miR interacted with varying concentrations of GQDs. (C) Fluorescent intensity of GQDs/FAM-miR in the solution after incubating with different concentrations of CTS-*CCND1* (200, 300, 400, 500, 600 and 700 nM). (D) Fluorescent recovery efficiency calculated to obtain the required ratio of CTS-*CCND1* for miR-193a-3p release in GQDs/FAM-miR.**Figure S4.** Fluorescence response of only GQDs/FAM-miR (No target), and GQDs/FAM-miR upon recognition of CTS-*CCND1* as well as scramble sequence. **Figure S5.** Characterization of human umbilical cord mesenchymal stem cells (hucMSCs). (A) Surface antigen expression on hucMSCs detected by flow cytometry. HucMSCs expressed CD29, CD105, and CD73, but did not express CD11b, CD45 and CD34 [2]. (B) Images showing the undifferentiated hucMSCs and MSCs that had adipocytic or osteocytic differentiation capacity. **Figure S6.** (A) TEM images and (B) Size measurements of hucMSCs-derived sEVs treated by different sonication parameters. **Figure S7.** The subcellular localization of CCND1 mRNA are nuclei and cytoplasm (white arrows) detected by RNA-FISH, where scale bar = 100 μm. **Figure S8.** FTIR spectra of GQDs. It contains the–NH, C=C, C=N/C=O and C–H groups. **Figure S9.** CCK-8 assay showed that different concentrations of GQDs/Cy5-miR@sEVs inhibited HGC-27 cell viability. Data are shown as mean ± SD and analyzed by one-way ANOVA. *p<0.05, ****p<0.0001.**Figure S10.** (A) Transwell migration assays and (B) matrigel invasion assays of HGC-27 cells when treated with PBS, GQDs, sEVs, GQDs/Cy5-miR, and GQDs/Cy5-miR@sEVs, respectively. Data are expressed as mean ± SD and analyzed by one-way ANOVA. ***p<0.001.

## Data Availability

The data and materials used in this manuscript will be made available upon request to the corresponding authors.
