# Supplementary material for: Visualization of microRNA therapy in cancers delivered by small extracellular vesicles
Source: J Nanobiotechnology. 2023 Nov 29;21:457. doi: 10.1186/s12951-023-02187-5 (PMC10685536; doi:10.1186/s12951-023-02187-5)
Supplement: Supplementary file 1 — Additional file 1: Table S1. Top 3 enriched Kyoto Encyclopedia of Genes and Genomes (KEGG) pathways for genes that were significantly downregulated by miR-193a-3p [1]. Figure S1. Identification of CCND1 as a target gene for miR-193a-3p delivery. (A) Venn diagram of miR-193a-3p target genes in three databases (PicTar, miRDB, TargetScan). (B) The expression of 29 target genes in tumor tissues and normal tissues according to TCGA and GTEx databases. The 18 underscored genes show higher expression in GC tissues than in normal tissues (T>N). (C) Venn diagram of two gene sets. Predicted target genes (T>N), a gene set that was upregulated in GC tissues identified by TCGA and GTEx databases. Cell-cycle category, a gene set that was maximally regulated by miR-193a-3p from GEO database. (D) qRT-PCR assays showing the mRNA expression of YWHAZ and CCND1 in GC cell lines (HGC-27, MKN-28, AGS, SNU-1) and a normal human gastric mucosal epithelial cell line (GES-1). (E) The combination of miR-193a-3p and its target gene CCND1. Figure S2. Preparation and characterization of GQDs and miR-193a-3p. (A) HR-TEM images of GQDs. (B) UV–Vis absorption of GQDs from 200 to 800 nm. (C) Emissions spectra of GQDs when excited at various wavelengths in dilute aqueous solutions. (D) Fluorescent intensity of GQDs in the concentration of 2, 5, 10, 20, 30, 40, and 50 μg/mL, respectively. (E) Fluorescent intensity of Cy5-miR in the concentration of 20, 50, 100, 200, 300, 400, and 500 nM, respectively. (F) Fluorescent intensity of FAM-miR with the concentration of 20, 50, 100, 200, 350, and 500 nM, respectively. Figure S3. The interactions between the FAM-tagged miRNAs and GQDs. (A) Fluorescent intensity of FAM-miR (100 nM) in the presence of varying concentrations of GQDs (2, 6, 8, 10, 20, 30, 40, 50, 60, 70 and 80 μg/mL) (B) The fluorescent quenching efficiency of FAM-miR (100 nM) and GQDs was calculated. F0: Fluorescent intensity of FAM-miR without GQDs. F: Fluorescent intensity of the FAM-miR inter [file 12951_2023_2187_MOESM1_ESM.docx]

**Additional file 1**

**for**

**Visualization of microRNA therapy in cancers delivered by small extracellular vesicles**

Peiwen Fu^‡, 1, 2^, Yumeng Guo^‡, 1^, Yanan Luo^1^, Michael Mak^3^, Jianguo Zhang^4^, Wenrong Xu^1, 5, *^, Hui Qian^1, 5, *^, Zhimin Tao^1, 3, 4, 5, *^

^1^Jiangsu Province Key Laboratory of Medical Science and Laboratory Medicine, Department of Laboratory Medicine, School of Medicine, Jiangsu University, Zhenjiang, Jiangsu 212013, China

^2^Department of Laboratory Medicine, Nanjing First Hospital, Nanjing Medical

University, Nanjing, Jiangsu 210006, China

^3^Department of Biomedical Engineering, School of Engineering and Applied Science, Yale University, New Haven 06520, USA

^4^Department of Emergency Medicine, The Affiliated Hospital, Jiangsu University, Zhenjiang, Jiangsu 212001, China

^5^Zhenjiang Key Laboratory of High Technology Research on Exosomes Foundation and Transformation Application, School of Medicine, Jiangsu University, Zhenjiang, Jiangsu 212013, China

^‡^These authors contributed equally to this work.

^*^Correspondences should be addressed to:

Wenrong Xu, [icls@ujs.edu.cn](mailto:icls@ujs.edu.cn) (ORCID: 0000-0003-0903-1973)

Hui Qian, [lstmmmlst@163.com](mailto:lstmmmlst@163.com) (ORCID: 0000-0002-0098-3196)

Zhimin Tao, [jsutao@ujs.edu.cn](mailto:jsutao@ujs.edu.cn) (ORCID: 0000-0002-9765-2720)

**Table S1.** Top 3 enriched Kyoto Encyclopedia of Genes and Genomes (KEGG) pathways for genes that were significantly downregulated by miR-193a-3p[1].


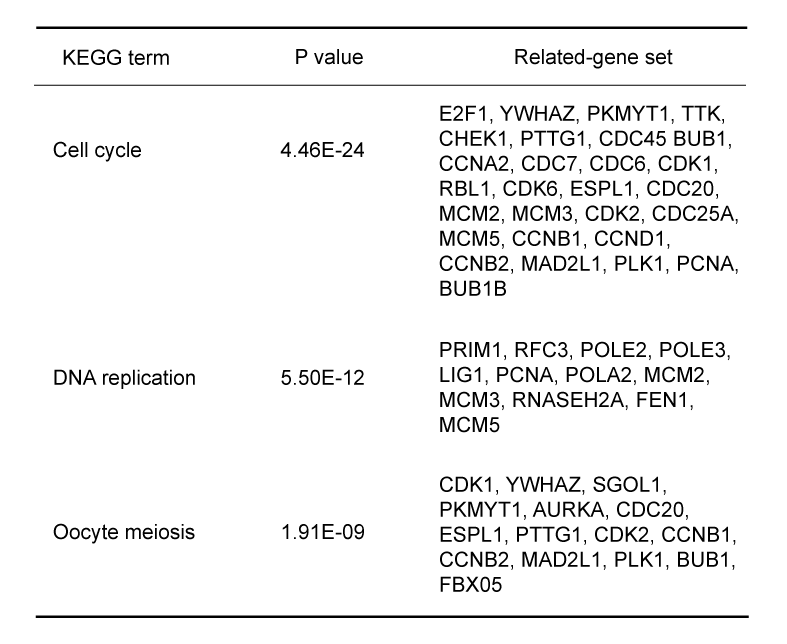


**Figure S1**


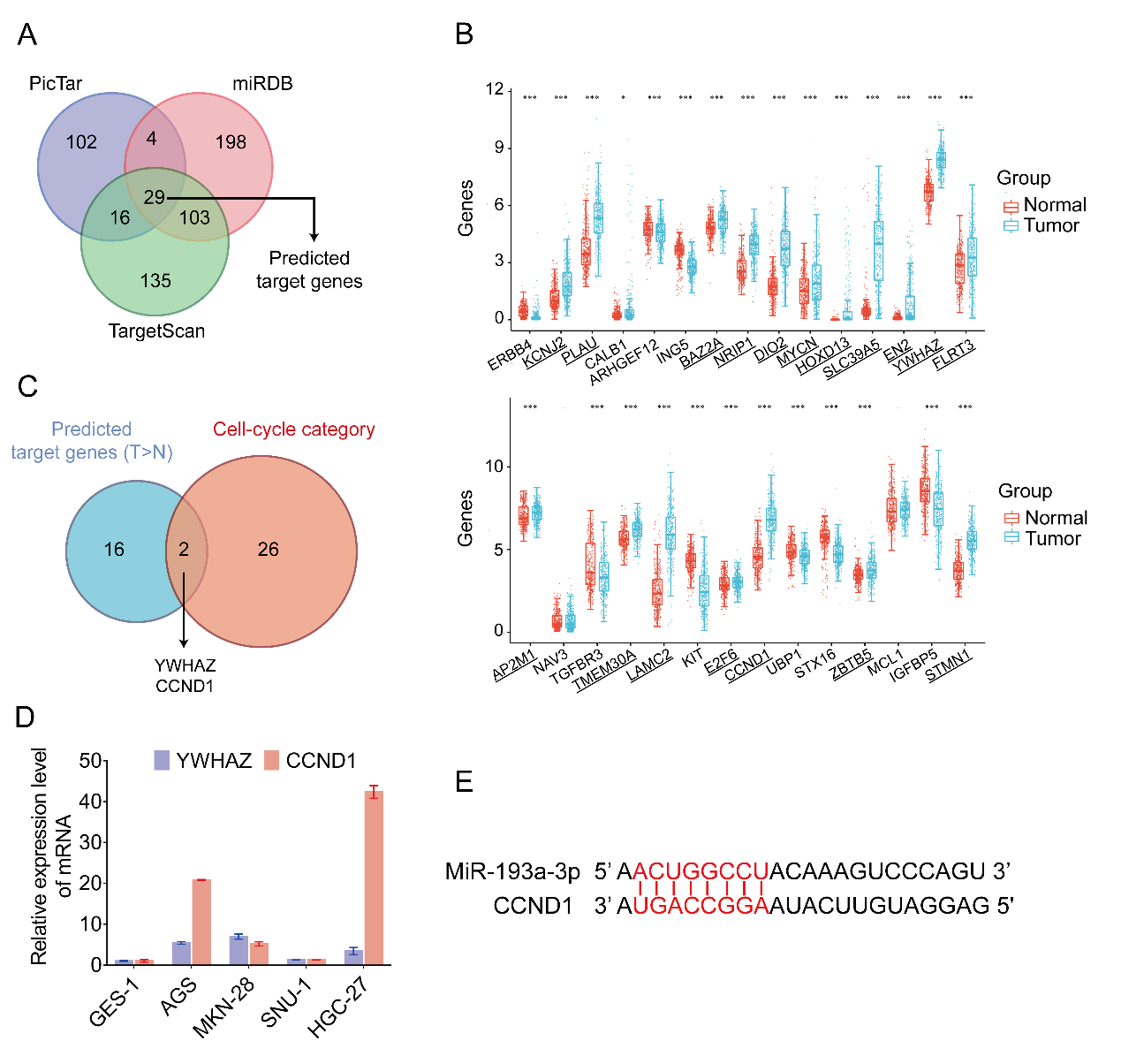


**Figure S1.** Identification of *CCND1* as a target gene for miR-193a-3p delivery. (A) Venn diagram of miR-193a-3p target genes in three databases (PicTar, miRDB, TargetScan). (B) The expression of 29 target genes in tumor tissues and normal tissues according to TCGA and GTEx databases. The 18 underscored genes show higher expression in GC tissues than in normal tissues (T>N). (C) Venn diagram of two gene sets. Predicted target genes (T>N), a gene set that was upregulated in GC tissues identified by TCGA and GTEx databases. Cell-cycle category, a gene set that was maximally regulated by miR-193a-3p from GEO database. (D) qRT-PCR assays showing the mRNA expression of *YWHAZ* and *CCND1* in GC cell lines (HGC-27, MKN-28, AGS, SNU-1) and a normal human gastric mucosal epithelial cell line (GES-1). (E) The combination of miR-193a-3p and its target gene *CCND1*.

**Figure S2**


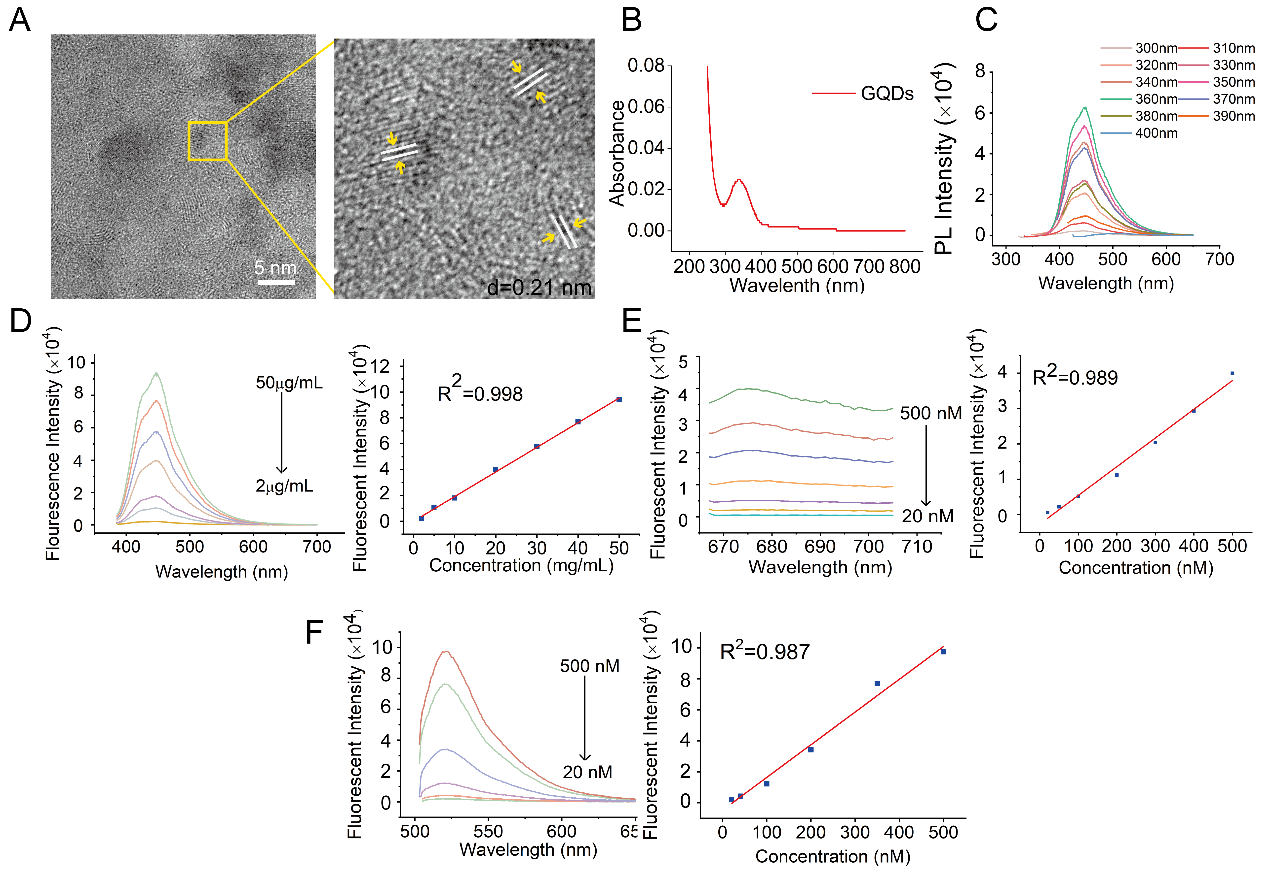


**Figure S2.** Preparation and characterization of GQDs and miR-193a-3p. (A) HR-TEM images of GQDs. (B) UV–Vis absorption of GQDs from 200 to 800 nm. (C) Emissions spectra of GQDs when excited at various wavelengths in dilute aqueous solutions. (D) Fluorescent intensity of GQDs in the concentration of 2, 5, 10, 20, 30, 40, and 50 μg/mL, respectively. (E) Fluorescent intensity of Cy5-miR in the concentration of 20, 50, 100, 200, 300, 400, and 500 nM, respectively. (F) Fluorescent intensity of FAM-miR with the concentration of 20, 50, 100, 200, 350, and 500 nM, respectively.

**Figure S3**


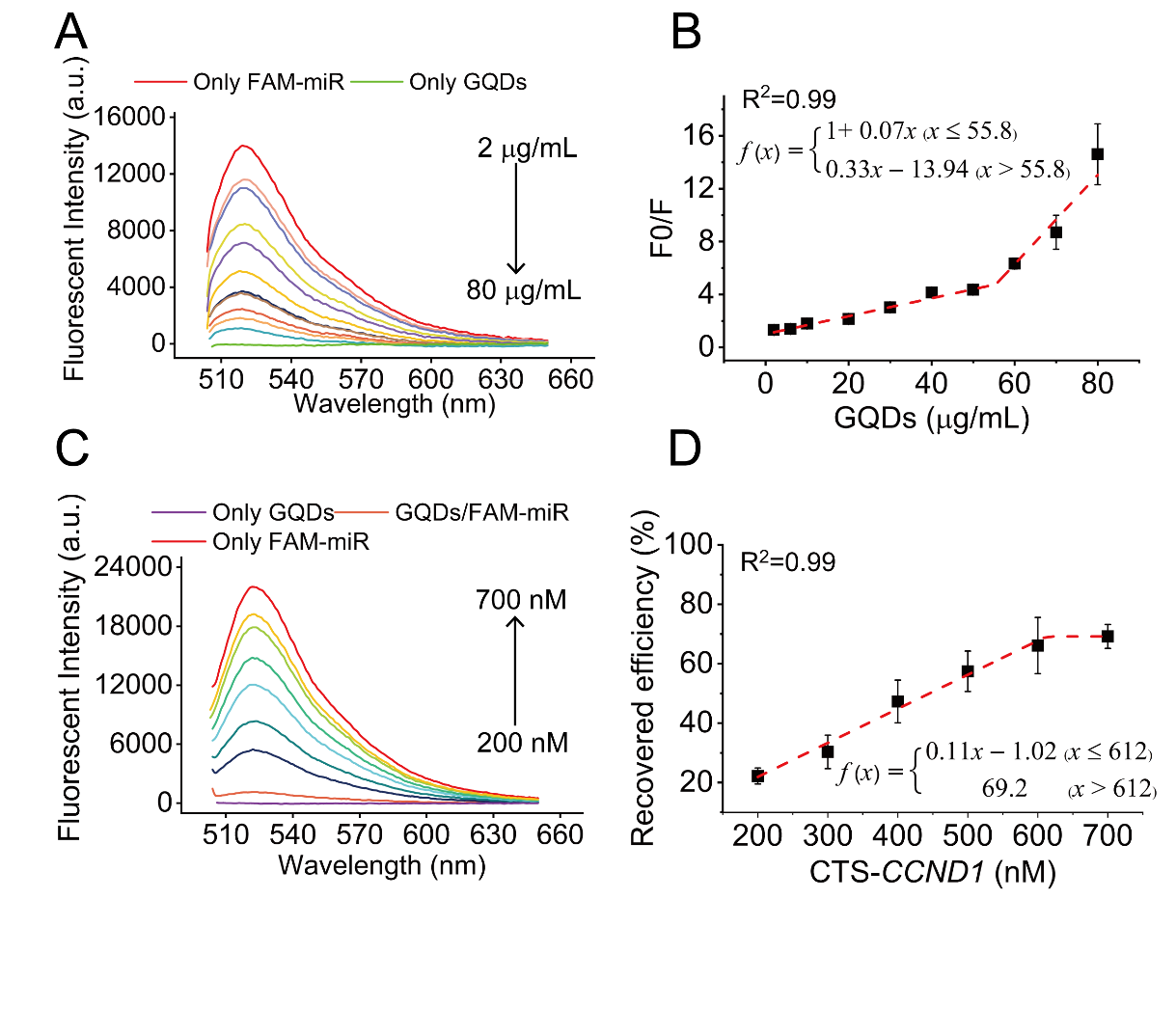


**Figure S3.** The interactions between the FAM-tagged miRNAs and GQDs. (A) Fluorescent intensity of FAM-miR (100 nM) in the presence of varying concentrations of GQDs (2, 6, 8, 10, 20, 30, 40, 50, 60, 70 and 80 μg/mL) (B) The fluorescent quenching efficiency of FAM-miR (100 nM) and GQDs was calculated. F0: Fluorescent intensity of FAM-miR without GQDs. F: Fluorescent intensity of the FAM-miR interacted with varying concentrations of GQDs. (C) Fluorescent intensity of GQDs/FAM-miR in the solution after incubating with different concentrations of CTS-*CCND1* (200, 300, 400, 500, 600 and 700 nM). (D) Fluorescent recovery efficiency calculated to obtain the required ratio of CTS-*CCND1* for miR-193a-3p release in GQDs/FAM-miR.

**Figure S4**





**Figure S4.** Fluorescence response of only GQDs/FAM-miR (No target), and GQDs/FAM-miR upon recognition of CTS-*CCND1* as well as scramble sequence.

**Figure S5**


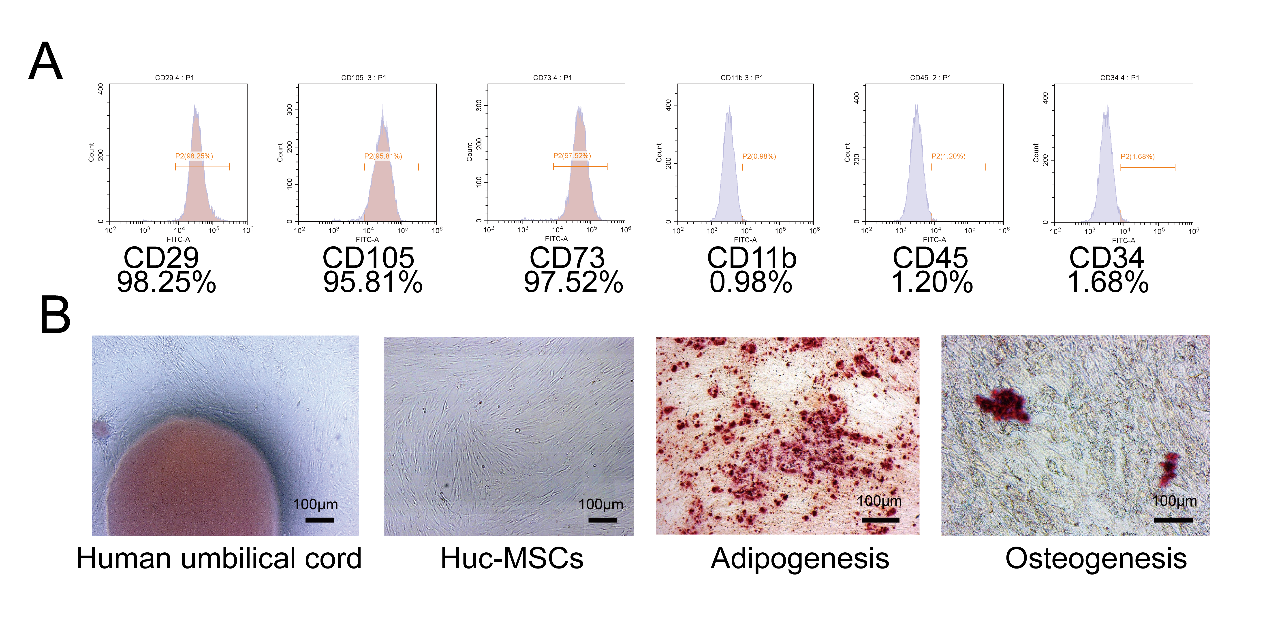


**Figure S5.** Characterization of human umbilical cord mesenchymal stem cells (hucMSCs). (A) Surface antigen expression on hucMSCs detected by flow cytometry. HucMSCs expressed CD29, CD105, and CD73, but did not express CD11b, CD45 and CD34[2]. (B) Images showing the undifferentiated hucMSCs and MSCs that had adipocytic or osteocytic differentiation capacity.

**Figure S6**


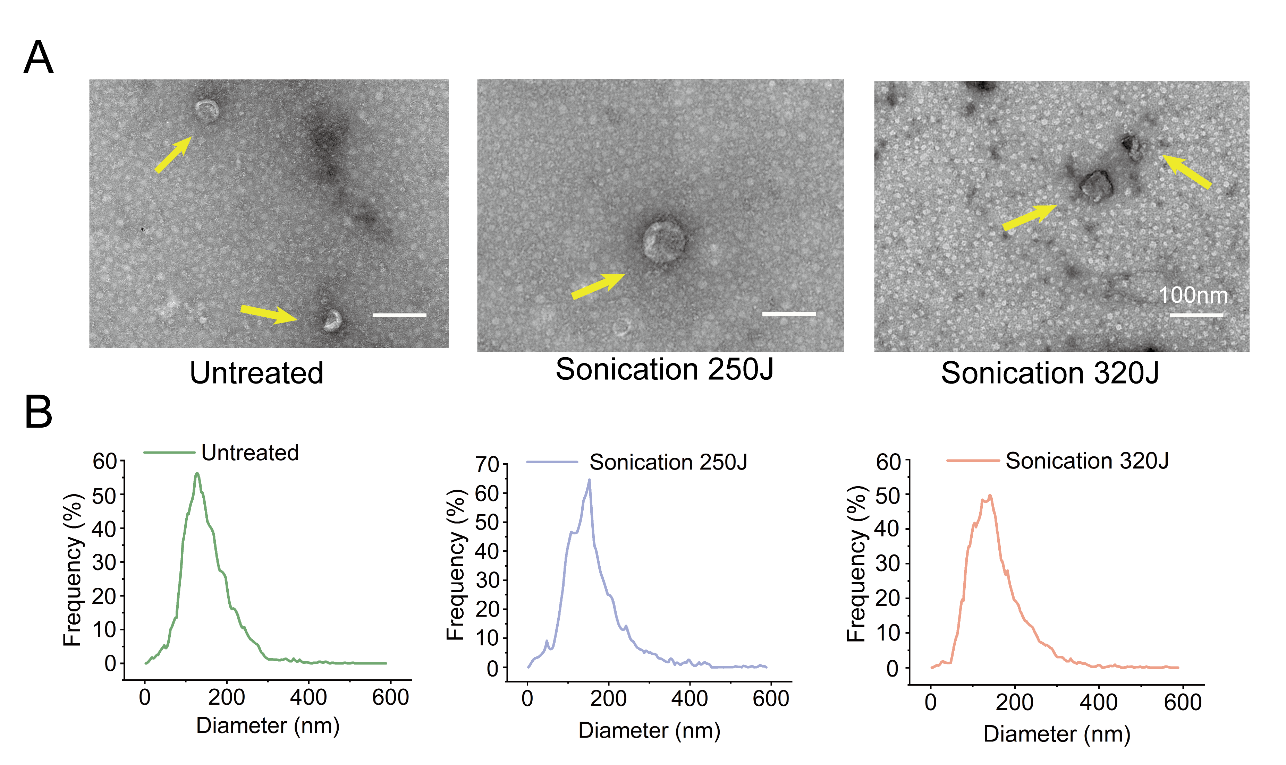


**Figure S6.** (A) TEM images and (B) Size measurements of hucMSCs-derived sEVs treated by different sonication parameters.

**Figure S7**
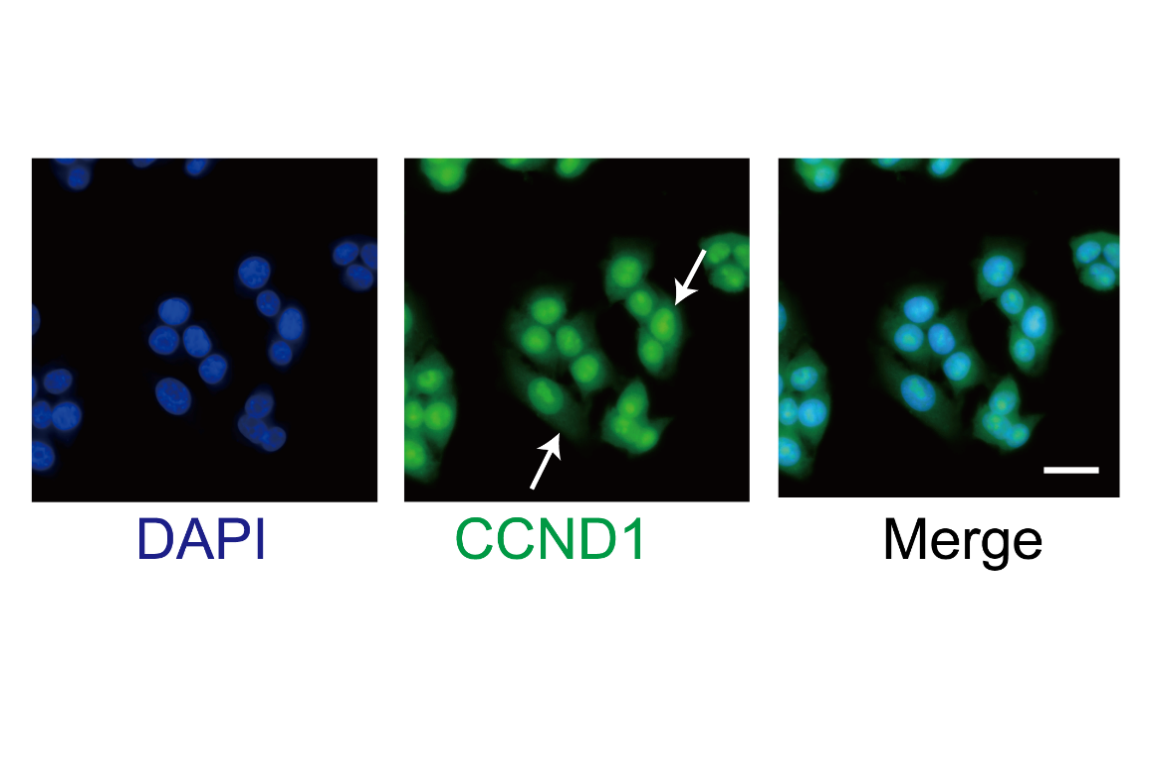


**Figure S7.** The subcellular localization of CCND1 mRNA are nuclei and cytoplasm (white arrows) detected by RNA-FISH, where scale bar = 100 μm.

**Figure S8**


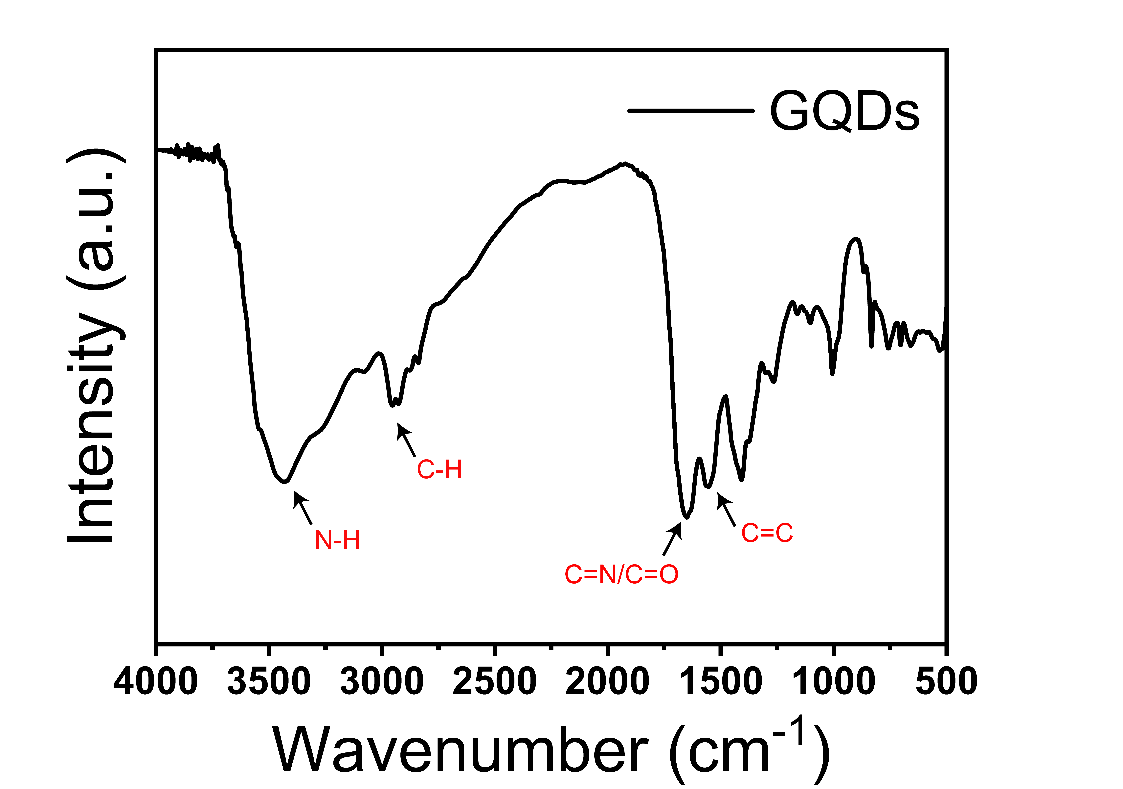


**Figure S8.** FTIR spectra of GQDs. It contains the -NH, C=C, C=N/C=O and C-H groups.

**Figure S9**


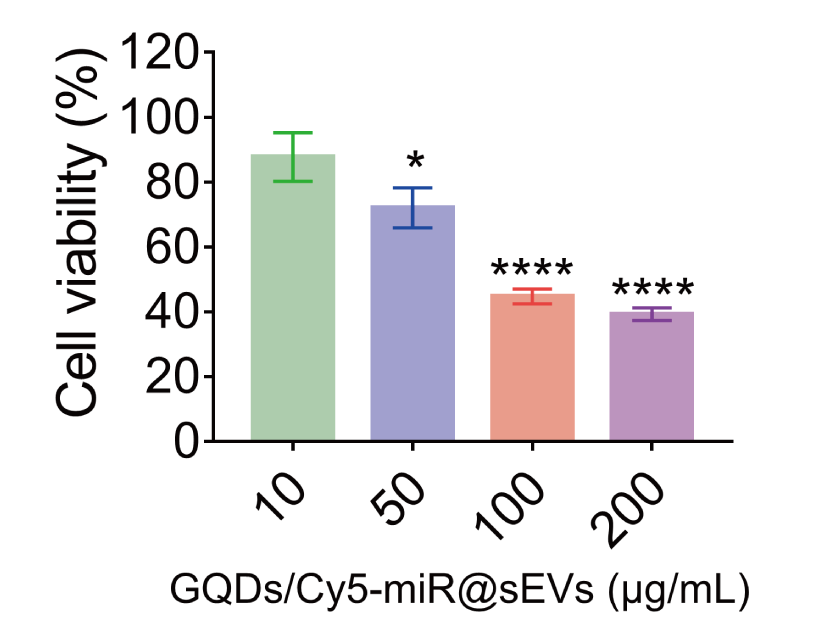


**Figure S9.** CCK-8 assay showed that different concentrations of GQDs/Cy5-miR@sEVs inhibited HGC-27 cell viability. Data are shown as mean ± SD and analyzed by one-way ANOVA. **p*<0.05, *****p*<0.0001.

**Figure S10**


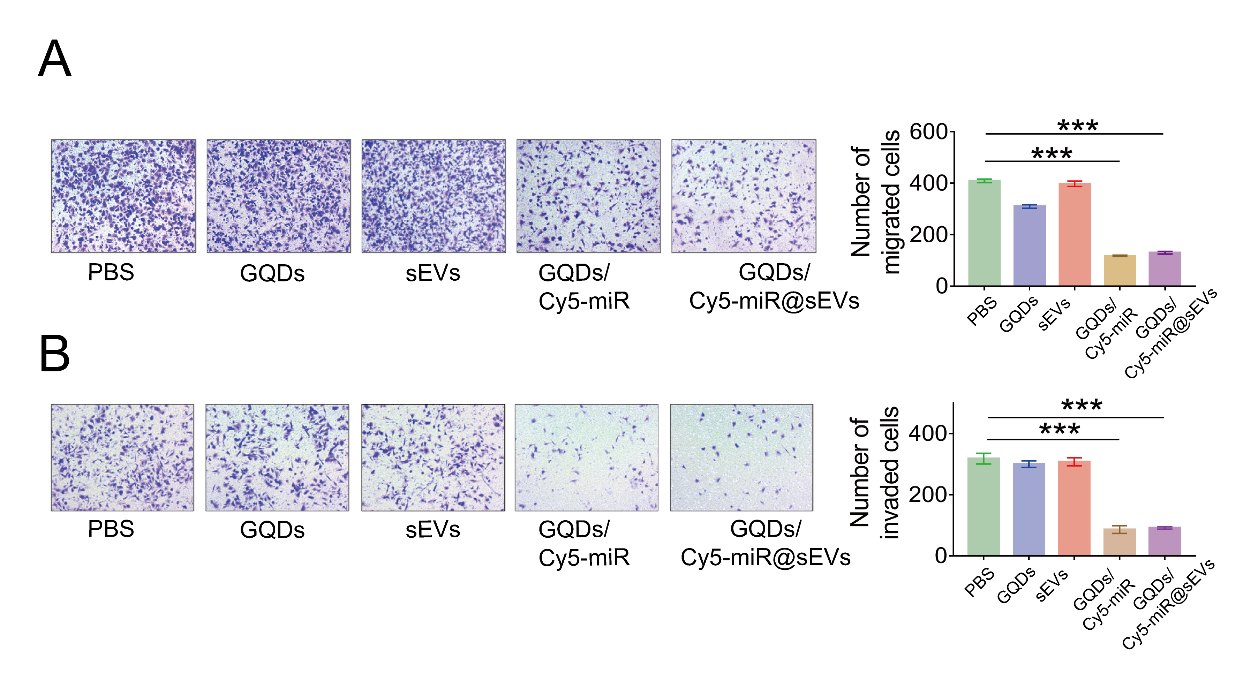


**Figure S10.** (A) Transwell migration assays and (B) matrigel invasion assays of HGC-27 cells when treated with PBS, GQDs, sEVs, GQDs/Cy5-miR, and GQDs/Cy5-miR@sEVs, respectively. Data are expressed as mean ± SD and analyzed by one-way ANOVA. ****p*<0.001.

**References**

[1] P. Hydbring, Y. Wang, A. Fassl, X. Li, V. Matia, T. Otto, Y.J. Choi, K.E. Sweeney, J.M. Suski, H. Yin, R.L. Bogorad, S. Goel, H. Yuzugullu, K.J. Kauffman, J. Yang, C. Jin, Y. Li, D. Floris, R. Swanson, K. Ng, E. Sicinska, L. Anders, J.J. Zhao, K. Polyak, D.G. Anderson, C. Li, P. Sicinski, Cell-Cycle-Targeting MicroRNAs as Therapeutic Tools against Refractory Cancers, Cancer Cell 31(4) (2017) 576-590.e8.

[2] M.S. Divya, G.E. Roshin, T.S. Divya, V.A. Rasheed, T.R. Santhoshkumar, K.E. Elizabeth, J. James, R.M. Pillai, Umbilical cord blood-derived mesenchymal stem cells consist of a unique population of progenitors co-expressing mesenchymal stem cell and neuronal markers capable of instantaneous neuronal differentiation, Stem Cell Res Ther 3(6) (2012) 57.
